# Temporal comorbidity patterns in Alzheimer's disease and vascular dementia: A population‐based observational study in UK Biobank

**DOI:** 10.1002/dad2.70265

**Published:** 2026-02-10

**Authors:** Chloe Walsh, Antigone Fogel, Ann‐Kathrin Schalkamp, Vanessa Mabiala, Behnam Shariati, Cynthia Sandor, Mina Ryten, Ramin Nilforooshan, Payam Barnaghi

**Affiliations:** ^1^ Department of Brain Sciences Imperial College London London UK; ^2^ Surrey and Borders Partnership NHS Foundation Trust Leatherhead UK; ^3^ UK Dementia Research Institute Care Research & Technology Centre Guildford UK; ^4^ UK Dementia Research Institute Imperial College London UK; ^5^ Bakar Computational Health Sciences Institute, University of California San Francisco USA; ^6^ UK Dementia Research Institute Cambridge Cambridge UK; ^7^ Department of Clinical Neurosciences University of Cambridge Cambridge UK; ^8^ Department of Genomic Medicine University of Cambridge Cambridge UK; ^9^ Faculty of Health and Medical Sciences University of Surrey Guildford United Kingdom

**Keywords:** Alzheimer's disease, chronological mapping, comorbidities, longitudinal, machine learning, UK Biobank, vascular dementia

## Abstract

**Introduction:**

In Alzheimer's disease (AD) and vascular dementia (VaD), comorbidities shape disease trajectories and care needs, yet their timing across the lifespan remains poorly understood.

**Methods:**

We analyzed comorbidities using in‐patient hospital International Classification of Diseases 10th Revision codes in 10,730 UK Biobank participants with AD or VaD, spanning 20 years before to 10 years after diagnosis. Logistic regression and Bayesian network analysis identified time‐ and subtype‐specific risk patterns, validated against controls.

**Results:**

Distinct comorbidities emerged decades before diagnosis. In AD, depressive episodes, osteoporosis, and type 1 diabetes appeared up to 20 years pre‐diagnosis, while VaD was characterized by early cerebral infarctions, type 1 diabetes, intestinal disorders, and rheumatoid arthritis, absent in controls.

**Discussion:**

Although restricted to severe populations captured in in‐patient data, excluding primary care, these findings reveal time‐dependent prodromal patterns in AD and VaD, highlighting opportunities for targeted screening, prevention, and early intervention.

## Highlights

- Distinct comorbidity patterns emerge up to 20 years before dementia diagnosis.
- Alzheimer's disease (AD) and vascular dementia (VaD) show unique, time‐specific risk profiles.
- Comorbidities such as depression and osteoporosis are highly associated with AD.
- VaD was linked to cerebrovascular and inflammatory conditions before symptom onset.
- Findings support targeted screening and early, subtype‐specific interventions.

## 1 BACKGROUND

Comorbidities play a critical role in both the development and progression of dementia.^1^, ^2^ Several decades before clinical onset, long‐term conditions such as hypertension, depression, and metabolic disorders develop, contributing to an increased risk of dementia later in life. After diagnosis, comorbidities further compound the disease burden, worsening quality of life and increasing health needs. Yet, approximately half of dementia risk remains unexplained by known lifestyle, behavioral, socioeconomic, or genetic factors.^3^ The Lancet Commission has identified modifiable risk factors for dementia prevention and care,^4^, ^5^, ^6^ and recent work increasingly supports the view of dementia as a multisystem condition.^7^, ^8^ However, most studies have examined isolated comorbidities, without consideration of their timing, interactions, or subtype specificity.^9^

Dementia comprises multiple subtypes, including Alzheimer's disease (AD) and vascular dementia (VaD), each with distinct pathophysiological signatures.^10^ Emerging evidence shows that these subtypes are characterized by different comorbidity profiles, which may influence the speed of decline, mortality, and functional outcomes.^11^, ^12^, ^13^ While some longitudinal studies have identified early associations between conditions and dementia, few have included the timing or temporal dynamics of comorbidities.^14^ One recent study reported musculoskeletal and sensory conditions preceding AD, and cerebrovascular diseases preceding VaD, up to 5 years before diagnosis; this work was limited by a small sample size and short follow‐up window.^15^ Additionally, recent findings around comorbidities in AD and VaD have implications for clinical practice, suggesting that dementia assessment and management should account for broader physical and mental health profiles rather than focusing exclusively on cognitive symptoms.^16^ The overlapping comorbidity trajectories observed across AD and VaD groups lend support to mixed pathology models and highlight limitations of categorical diagnostic frameworks in older populations.

Recent works have contributed substantially to this field by using dynamic time warping and clustering methods to identify diagnostic trajectories preceding AD from electronic health records.^17^ These findings highlighted meaningful diagnostic sequences and offered early causal insights. Building on this work, our study expands both the methodological and clinical scope by including VaD and quantifying the relative risk associated with each comorbidity across specific time windows. Instead of grouping conditions into general trajectories, we model the temporal evolution of comorbidities and their subtype‐specific risk associations, enabling more precise early detection and risk stratification.

To address current limitations, we analyzed electronic hospital records from the UK Biobank spanning 20 years before and 10 years after dementia diagnosis. By comparing AD and VaD, and age‐ and sex‐matched controls, we generated temporal comorbidity profiles across the entire diagnostic window. This allowed us to identify distinct early patterns, such as osteoporosis and irritable bowel syndrome in AD, and cerebral infarctions and type 1 diabetes in VaD, not observed in controls or the other dementia subtype. Our approach captures the pattern of multi‐morbidities over time that are unique to AD and VaD. Shifting the perspective of dementia risk management, the timeline of comorbidities identified present more targeted windows of opportunity.

## 2 METHODS

### 2.1 Study design and data processing

**RESEARCH IN CONTEXT**

1. **Systematic review**: We reviewed PubMed, Embase, and Web of Science using combinations of terms relating to dementia subtypes, comorbidities, temporality, and electronic health records. Previous large‐scale studies show that individuals who develop dementia experience an increased burden of long‐term conditions many years before diagnosis and exhibit comorbidity patterns distinct from cognitively healthy aging populations. However, existing work largely focuses on cumulative disease burden or grouped trajectory methods, often concentrates on Alzheimer's disease (AD), excludes vascular dementia, and uses limited observation windows, restricting insight into long‐term temporal dynamics.
2. **Interpretation**: We identified early, subtype‐specific comorbidity signatures, including osteoporosis and depressive episodes in AD, and cerebral infarctions and type 1 diabetes in vascular dementia, which were not observed in the alternative subtype or control group.
3. **Future directions**: High‐resolution, time‐specific mapping of individual conditions can reveal subtype‐specific risk signals decades before diagnosis, enabling more precise screening, stratified prevention strategies, and scalable public health approaches using longitudinal clinical data.

This cohort study used data from the UK Biobank, a large biomedical database of 446,848 UK participants aged 40 to 73 assessed between 2006 and 2010.^18^ This age range reflects the UK Biobank recruitment limits, as opposed to the age range of the data presented. Participants provided biological samples, completed questionnaires, and underwent physical examinations. Hospital inpatient records, coded using a standardized system of World Health Organization (WHO) International Classification of Disease 10th Revision (ICD‐10) codes (further detailed in Table S1 and Figure S1 in supporting information), were available for 446,814 participants and included only first‐instance diagnoses. Analysis was limited to inpatient records, as many AD and VaD cases lacked primary care data.

The UK Biobank electronic health‐care record data were collected after informed consent obtained from all participants. The North West Multi‐centre Research Ethics Committee (MREC) and Research Tissue Bank (RTB) approved the scientific protocol and operational procedures (REC reference number: 16/NW/027) of the UK Biobank. Data for this study were obtained and research was conducted under the UK Biobank applications license numbers 69610 and 109607, respectively.

Baseline characteristics extracted from the UK Biobank dataset included sex, year of birth, ethnicity, and date of death (if applicable). Reasons for death were also available but were inconsistently recorded for our cohort and, therefore, not included in the analysis. The analysis was adjusted for age and sex, when appropriate.

#### 2.1.1 Dementia diagnosis

**FIGURE 1:**
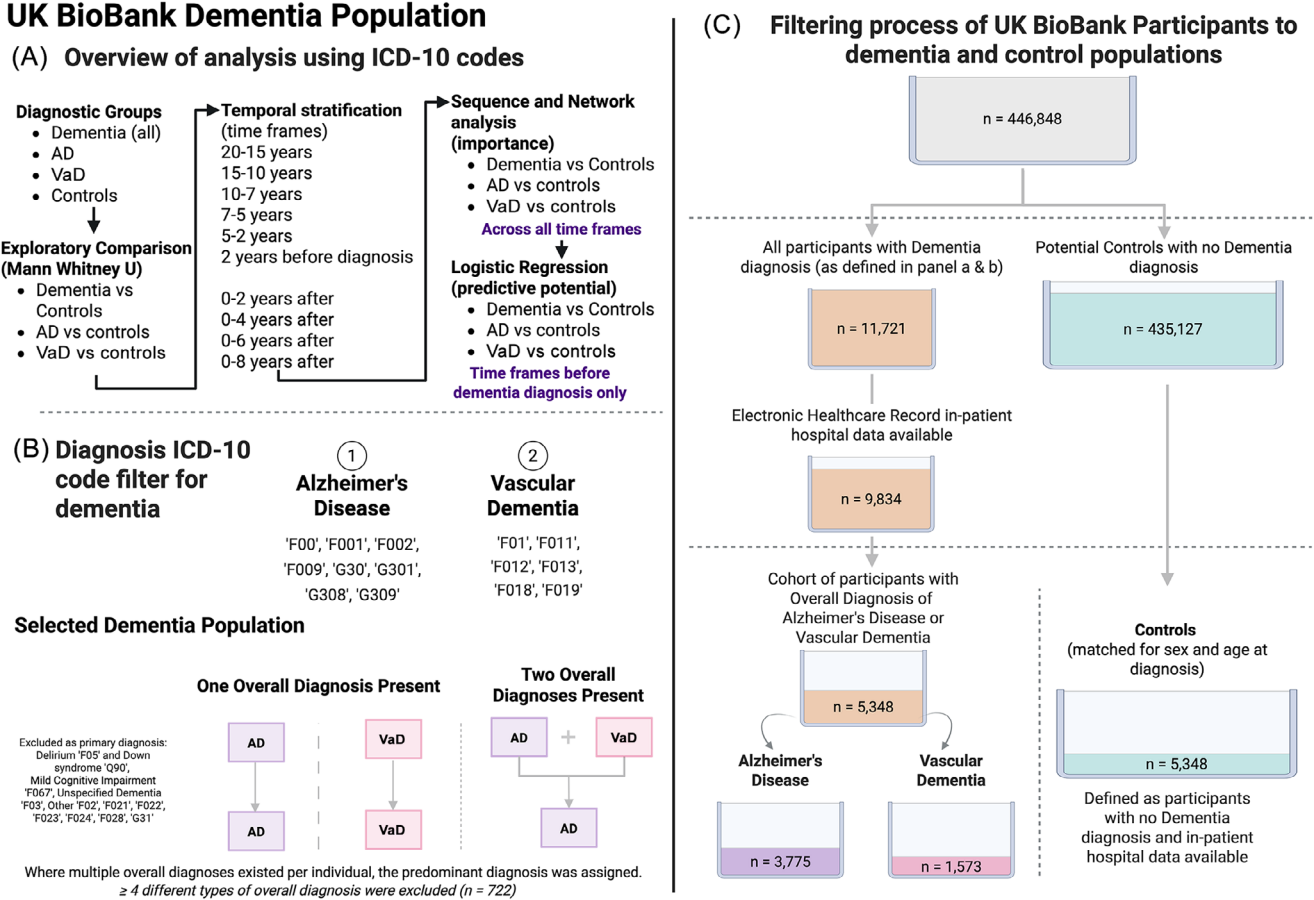
Schematic for the selection process of individuals from the UK Biobank dataset. A, Overview of analysis using ICD‐10 codes to determine most associated comorbidities with AD and VaD compared to controls. Multi‐layered analysis was conducted to examine comorbidities from different analytic perspectives. B, Diagnosis ICD‐10 code filter for dementia—ICD‐10 codes used to identify potential dementia diagnoses and their assignment to overall diagnosis. Participants with multiple dementia subtype codes were classified as either AD or VaD, according to the most common overall diagnosis recorded. C, Filtering process of UK BioBank participants to dementia and control populations. Final cohort composition, including dementia subtypes and matched controls. Figure created with BioRender. AD, Alzheimer's disease; ICD‐10, International Classification of Diseases, 10th Revision; VaD, vascular dementia.

From the UK Biobank cohort, individuals had multiple diagnoses of dementia subtypes; therefore, we established a workflow to distinguish the most likely diagnosis (Figure 1). This process was similar to other work in the Scottish sub‐set of the UK Biobank population, in which coded diagnoses were compared across multiple primary care, inpatient, and mortality data, with review by clinicians and dementia experts.^19^ Further details are provided in supporting information (Supplementary Methods—Section 4.1).

We first defined a subset of ICD‐10 codes to determine dementia diagnosis using ICD‐10 classification, version 2019.^20^ Codes for AD were: F00, F001, F002, F009, G30, G301, G308, and G309; codes for VaD were: F01, F011, F012, F013, F018, and F019; codes for “Other” were: F02, F021, F022, F023, F024, F028, and G31; “Unspecified Dementia” code was: F03; and for “Mild Cognitive Impairment” the code was: F067. We excluded delirium‐related conditions, code F05, that were not dementia diagnoses. This was only excluded as a primary diagnosis when filtering participants for the dementia population. Throughout the full analysis, any delirium conditions diagnosed as comorbidities within the electronic health record were retained and included in analysis. We also excluded the code Q90, corresponding to a diagnosis of Down syndrome (Supplementary Methods 4.2). This extracted 11,721 participants from the dataset. Participants who did not have in‐patient hospital data available were also excluded, resulting in a final cohort of 9834 potential participants (Figure 1). From the selected list of codes, we identified individuals with multiple dementia diagnoses in their records. Details of how participants were categorized according to their diagnosis can be found in supporting information (Supplementary Methods Section 4.3).

#### 2.1.2 Chronological mapping of comorbidities

Using “days to dementia diagnosis,” all ICD‐10 diagnoses of comorbidities were mapped relative to the central dementia diagnosis date (Supplementary Methods Section 4.4). For controls, a placeholder diagnosis flag date was assigned based on their age‐ and sex‐matched dementia participant (Supplementary Methods Section 4.5 and 4.6). Mappings were grouped into categorical time frames. For those before dementia: 20+ years before, 15 to 20 years before, 10 to 15 years before, 7 to 10 years before, 5 to 7 years before, 2 to 5 years before, and 0 to 2 years before, reducing the time frame span, as it got closer to the diagnosis date. For after dementia diagnosis, these were categorized into four time frames: 0 to 2 years after, 0 to 4 years after, 0 to 6 years after, 0 to 8 years after, and 0 to 10 years after. More information on temporal stratification is provided in supporting information (Supplementary Methods Section 4.7). We identified a condition as “new” if it had not already been diagnosed in previous years. Comorbidities have an important cumulative effect over time, and thus, it is not suitable to consider conditions only according to the time frame in which they first occur. They must also be considered over the time after emergence and in relation to each other.

### 2.2 Statistical analysis and machine learning

**FIGURE 2:**
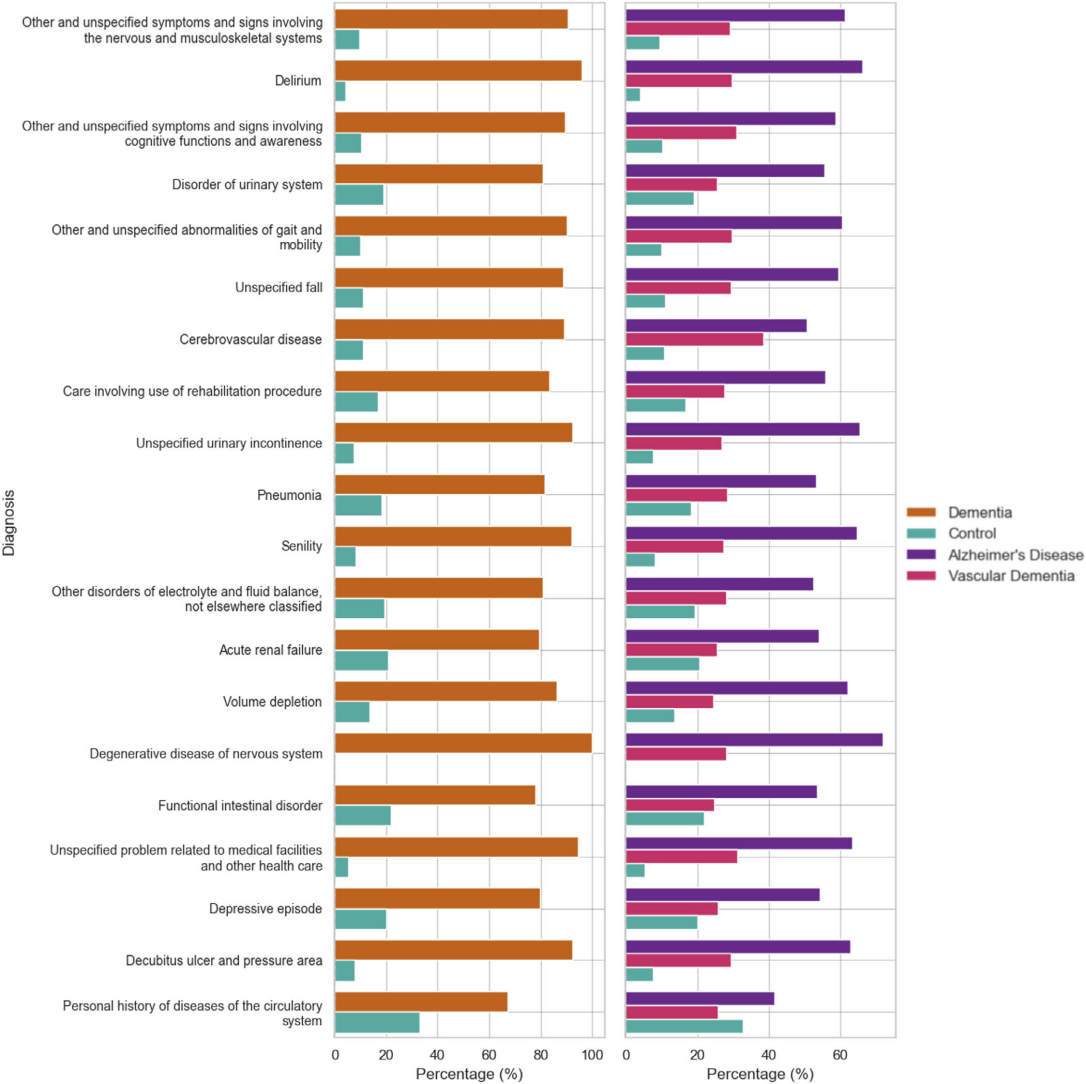
Prevalence of top 20 ICD‐10 conditions that were significantly different between dementia and controls. Here, a Mann–Whitney *U* test (all adjusted *P* values < 0.05, Bonferroni correction) was deployed, to compare association of each condition, to each dementia subtype. The first panel (left) categorized by either controls or dementia cohort. The second panel (right) categorized by either controls or dementia subtypes AD and VaD. Conditions are ordered from top to bottom, in order of most to least significant. ICD‐10 codes are often named with “unspecified” after each condition name, when that condition refers to a more general diagnosis. For readability purposes, “unspecified” was removed from the condition names although the ICD‐10 code was unchanged. Comorbidities and complications are significantly associated with dementia including circulatory diseases, mental health episodes and complications such as infections. AD, Alzheimer's disease; ICD‐10, International Classification of Diseases, 10th Revision; VaD, vascular dementia.

**FIGURE 3:**
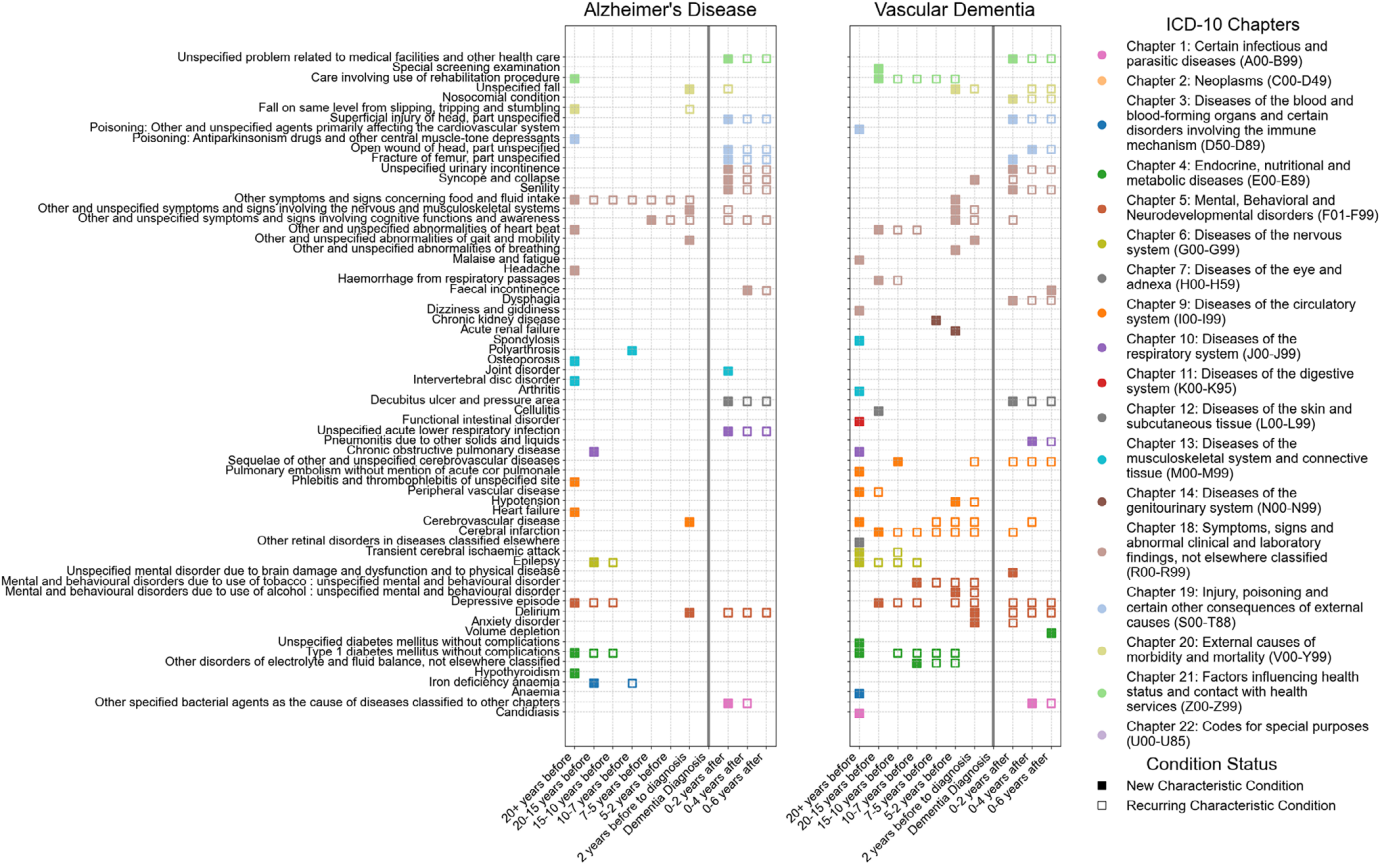
Chronological mapping of comorbidities associated with Alzheimer's disease (left panel), vascular dementia (center panel), from 20 years before diagnosis to 6 years after. Each time frame is shown, with the timing of dementia diagnosis marked by a solid vertical gray line. The conditions displayed represent individual types of ICD‐10 coded diagnoses. As detailed in the figure legend (“Condition Status”), filled color squares represent comorbidities that were newly characteristic of the cohort, at the given time frame, not seen in controls. Empty colored squares indicate comorbidities unique to the cohort that re‐occurred, in another timeframe. Colors correspond to higher‐level ICD‐10 Chapters, coded in the “ICD‐10 Chapters” legend. None of the conditions found to be characteristic of AD or VaD were characteristic of the cohort with hip fractures; therefore, only characteristic conditions are displayed here. For example, depressive episodes characteristic for both dementia subtype cohorts, were not characteristic of the cohort with hip fractures and therefore were unique to these disease populations. AD, Alzheimer's disease; ICD‐10, International Classification of Diseases, 10th Revision; VaD, vascular dementia.

**FIGURE 4:**
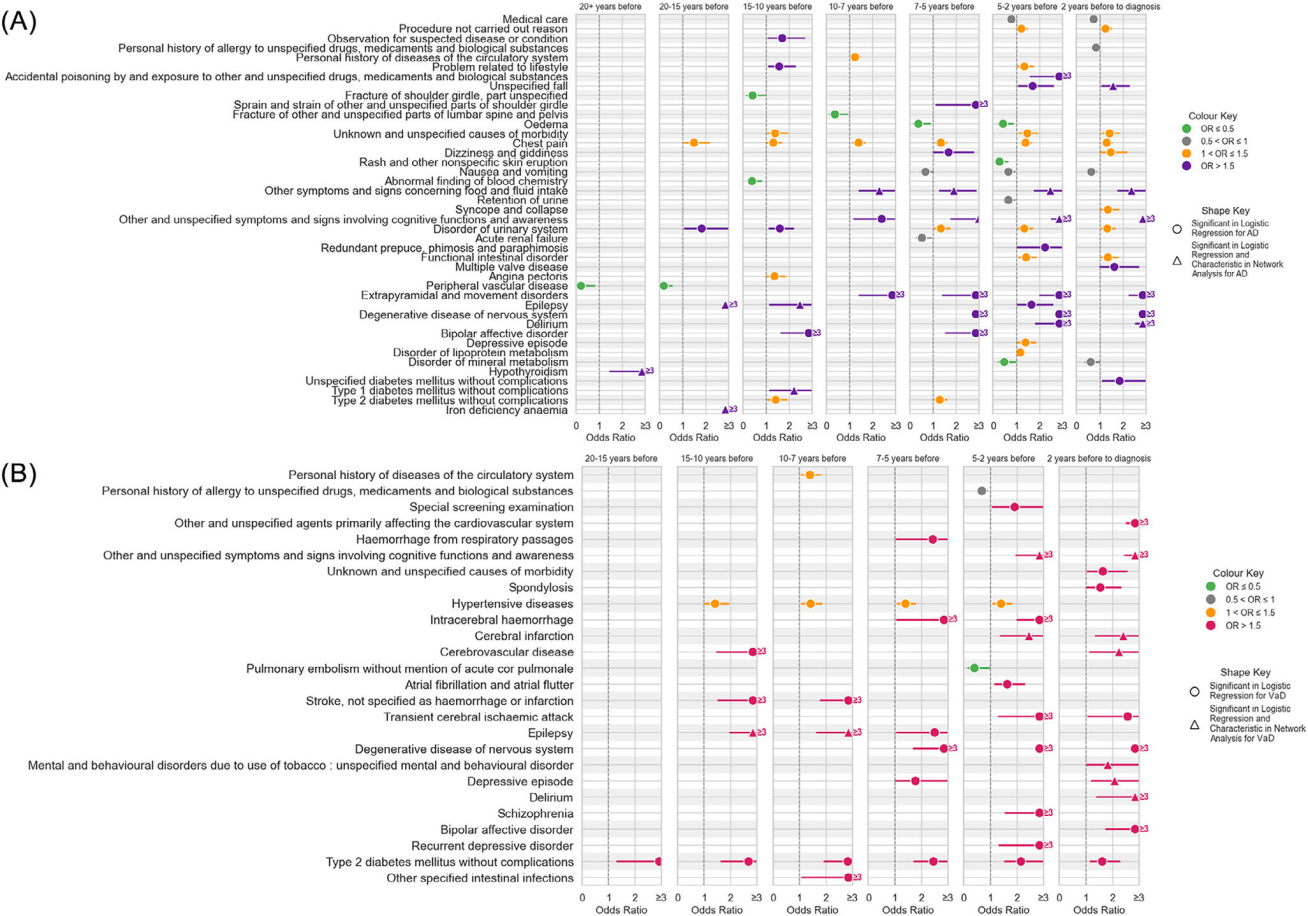
Significantly associated conditions and their ORs (with confidence intervals) for diagnosis of (A) AD and (B) VaD, adjusted for sex and age at dementia diagnosis. The logistic regression model included only conditions diagnosed prior to dementia diagnosis that also featured as significantly associated with dementia cohort, in Mann–Whitney *U* tests. This figure displays only conditions from the logistic regression model, with a significant association (*P* value < 0.05) with each dementia subtype. Data points refer to OR value and range of 95% confidence intervals. Depressive episodes, and complications related to food or fluid intake, show increasing association with AD from 20 years before diagnosis up to the point of diagnosis. Cerebral infarctions and cerebrovascular disease show consistently strong associations with VaD from 20 years before diagnosis up to the point of diagnosis. OR values of greater than three are plotted as “*>* 3.” AD, Alzheimer's disease; OR, odds ratio; VaD, vascular dementia.

**FIGURE 5:**
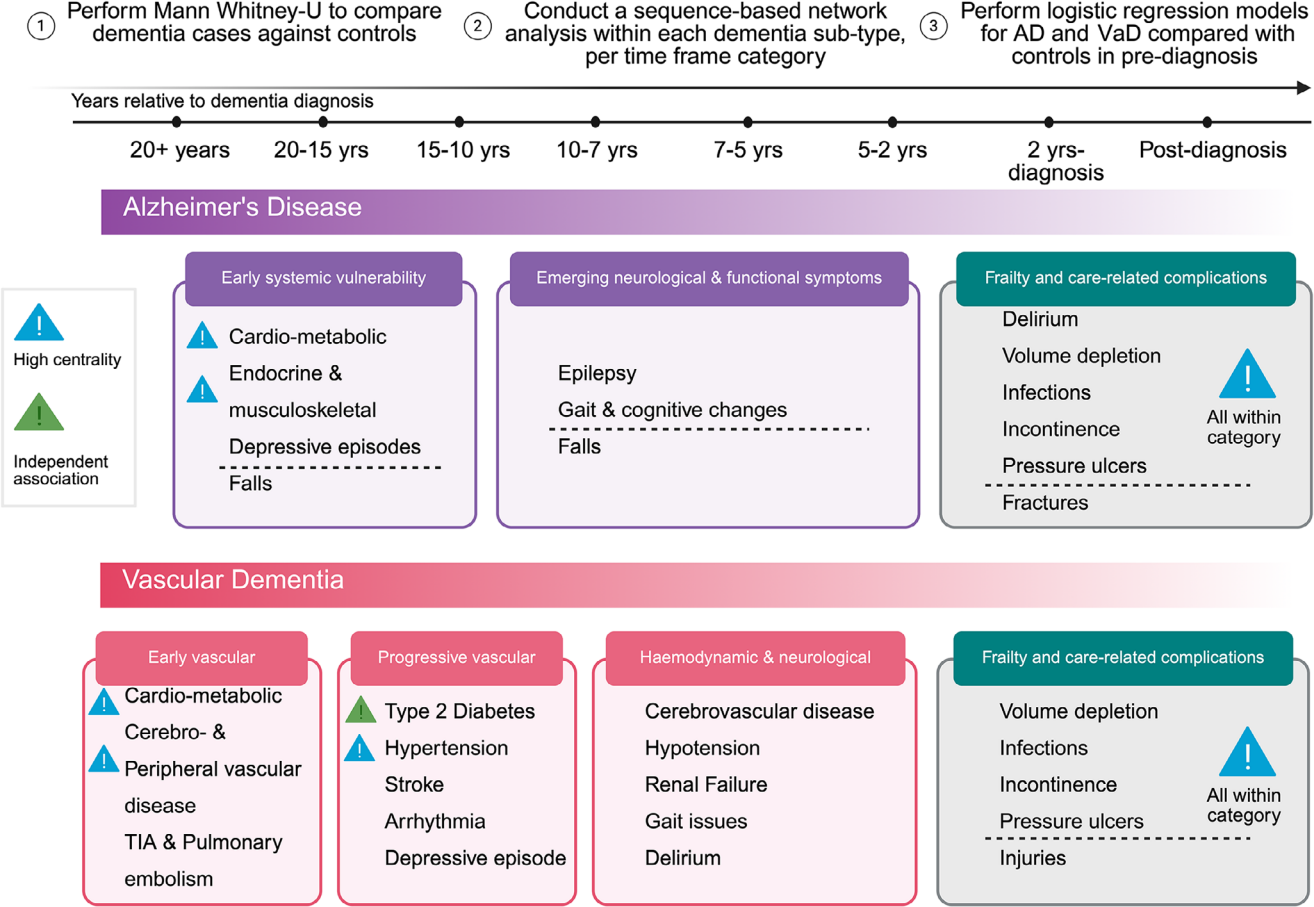
Overview of results across multi‐layered analysis. Summary of results from different elements of the analytical approaches conducted from Mann–Whitney testing globally, through to network analysis and logistic regression. Mann–Whitney: demonstrates global separation and breadth of systems involved. Networks identify when conditions become structurally important (central hubs) and what emerges “new” in each time frame. Regression quantifies independent associations per time frame and helps distinguish stable long‐term risk signals (e.g., diabetes/hypertension in VaD) from late‐stage syndrome or service‐use artifacts. Figure created with BioRender. AD, Alzheimer's disease; TIA, transient ischemic attack; VaD, vascular dementia.

An overview of analytical approaches is described fully in supporting information (Supplementary Methods Section 4.8). Here we summarize a brief overview of analytical approaches of the data presented. Figure 1 summarizes the analysis and how the population was selected based on ICD‐10 coding. Figure 2 depicts the significantly prominent conditions according to Mann–Whitney *U* tests (*P* value < 0.05), comparing dementia and controls overall. Figure 3 illustrates the sequence and network analysis^21^ results in a time‐dependent context (Supplementary Methods Section 4.9), to illustrate the most unique and significant conditions that were associated with that subtype, in each time frame, compared to controls and also compared to a control disease (hip fracture; Supplementary Methods Section 4.10). Figure 4 demonstrates the conditions found to be significantly associated with an outcome of either dementia subtype and their respective odds ratio, in each time frame. Importantly, those that shared a significance in this model as well as the initial statistical exploration of Mann–Whitney *U* testing are shown in a different shape (triangle). Finally, Figure 5 illustrates the overarching results and findings that were consistent across all analytical approaches and methods, specific to AD and VaD.

Polygenic risk scores (PRSs) for AD were compared between dementia subtypes and controls using Mann–Whitney *U* tests with Benjamini–Hochberg correction (see Supplementary Methods).^22^, ^23^ To assess the differences in prevalence of comorbidities between groups, we used the two‐sided Mann–Whitney *U* test,^24^ a non‐parametric test to compare two independent samples. We applied the Bonferroni correction^25^ to control for Type I errors; this was limited to correction across all comparisons between dementia and control groups. First, this analysis was conducted using individual diagnoses of ICD‐10 conditions, using a binary system for the presence or absence of the condition for each individual. A second analysis was conducted in parallel, using the sum of each ICD‐10 block per individual.

#### 2.2.1 Regression analyses of AD and VaD

Logistic regression models were run for each time frame, separately for AD and VaD, including age, age squared, and an age‐by‐sex interaction term. Model performance was assessed by area under the receiver operating characteristic curve. Various models were compared, including logistic regression with *_L_* 1 and *_L_* 2 regularization, Random Forest, and XGBoost. Odds ratios and confidence intervals were calculated, with significance set at *P *< 0.05.

### 2.3 Time to death analysis

Survival analysis was conducted, and hazard ratios were calculated using the Cox proportional hazards regression model^26^ with time to death as the dependent variable.

### 2.4 Role of the funding source

The funders of this study had no role in the design, set‐up, data collection, or analysis, nor did they have a role in writing of the report or the decision to submit the paper for publication. The corresponding author had full access to all the data in the study and had final responsibility for the decision to submit it for publication.

## 3 RESULTS

The UK Biobank population included 446,848 participants, with baseline assessments and in‐patient ICD‐10 codes available. Of 9834 participants with potential dementia diagnoses, 50% (*n* = 4874) had a diagnosis of one dementia subtype. Thirty percent (*n* = 2907) had two, 14% (*n* = 1331) had three, and 6% (*n* = 453) had four or more; participants with four or more different dementia subtype diagnoses were excluded (*n* = 722). For each participant, an overall mapping was established, as described in the section 2.1. Participants diagnosed with overall mapping of either unspecified dementia (*n* = 2114), mild cognitive impairment (*n* = 978), or other dementia diagnosis (*n* = 988), only, were excluded. The final cohort consisted of 5365 people diagnosed with dementia, who were then matched to a control group (*n* = 5365) using age at diagnosis and sex.

In the final cohort of 5365 participants with a dementia diagnosis, 3878 had AD subtype and 1487 had VaD subtype (Figure 1). A control cohort of 5365, who were matched to each dementia case based on age and sex, were included and defined as a population without a diagnosis of dementia in all of their in‐patient data (Supplementary Results section 4.11). This totaled 10,730 participants. Of the 5365 participants with dementia, 48% (*n* = 2583) were deceased, whereas in the control cohort, only 10% (*n* = 563) were deceased. Data regarding deceased participants was last updated in April 2024.

### 3.1 Comorbidities associated with dementia diagnosis

A Mann–Whitney *U* test was used to compare the comorbidities in those with dementia compared to their matched controls at the group level. Key comorbidities identified included circulatory and cerebrovascular disease, intestinal, and urinary or renal conditions. In addition, other consequential complications were reported in the dementia cohort, including pneumonia, decubitus ulcers and pressure areas (bed sores), delirium, and falls (*P* value < 0.05). The full list of significant conditions is included in Figure S2 in supporting information. The same analysis was conducted for block‐ or group‐level diagnoses of the ICD‐10 codes (Figure S3 in supporting information). The top 20 of the most significant ICD‐10 blocks are displayed in Figure 2, grouped by overall diagnosis.

### 3.2 Characteristic comorbidity analysis of AD and VaD

Considering dementia's systemic nature, we further analyzed the comorbidities that were most associated or characteristic to AD and VaD, described in section 3.1, using regression models to test independent association. Further details of model validation are found in supporting information (Supplementary Results section 4.12).

Logistic regression models, using only pre‐diagnosed conditions and adjusted for sex, age, and death date, established individual associations of comorbidities with AD or VaD (Figure 4). Model performance metrics are in Tables S2 and S3 in supporting information. Significant odds ratios (*P *< 0.05) are shown in Figure 4 and full results in Tables S4 and S5 in supporting information. For AD, key associated conditions included symptoms related to fluid and food intake (10–7 years pre‐diagnosis, *P *< 0.001) and delirium (5–2 years, *P *= 0.001). For VaD, cerebrovascular disease (15–10 years, *P *< 0.001) and intracerebral hemorrhage (7–5 years, *P *< 0.001) were most associated. Further details are detailed in supporting information (Supplementary Results 4.13).

### 3.3 Undirected network analysis of comorbidities associated with AD

For each dementia subtype, undirected network analysis was applied per time frame, across all time frames, for dementia and control cohorts (Figure 3), with degree centrality used to quantify each condition's relative importance among the others (Figure S4 in supporting information). A summary of the unique conditions is displayed in Figure 3.

Network ICD‐10 analyses per time frame (Figures S5–S6 in supporting information) showed that for AD, heart, circulatory, and hypertensive diseases became central (0.5–1.0) from 15–10 years pre‐diagnosis. For VaD, circulatory and heart diseases were central or highly influential pre‐diagnosis, but hypertensive diseases appeared later, and no new post‐diagnosis conditions unique to VaD were observed.

Characteristic conditions were identified for each subtype and time frame (Figure 3). Twenty years before AD, mental health conditions (e.g., depressive episodes), cardiovascular conditions (e.g., heart failure), and chronic illnesses (e.g., type 1 diabetes, hypothyroidism, osteoporosis) were prevalent, alongside events such as other symptoms involving food and fluid intake (Supplementary Results section 4.14). By 15 years pre‐diagnosis, additional conditions including iron deficiency anemia, epilepsy, and chronic obstructive pulmonary disease (COPD) were characteristic of AD. Considering more general diagnoses of conditions, using the ‘block’ level of ICD‐10 coding, metabolic conditions were also seen to preceed AD (Figure S7 in supporting information).

Post‐AD diagnosis, four new characteristic conditions were identified, including acute lower respiratory infections, bed sores, joint disorder, and bacterial agents as the cause of diseases from other ICD‐10 chapters as well as a range of signs, symptoms, and injuries (e.g., senility, syncope and collapse, head wounds, fractures and urinary incontinence), persisting for up to 6 years.

### 3.4 Undirected network analysis of comorbidities associated with VaD

Compared to AD, the VaD cohort showed a distinct profile (Figure 3). Twenty years before diagnosis, VaD was characterized by candidiasis, diabetes (type 1 and unspecified), epilepsy, transient cerebral ischemic attack, and retinal disorders, alongside cerebrovascular, peripheral vascular disease, COPD, functional intestinal disorders, arthritis, and spondylosis. By 15 years pre‐diagnosis, depressive episodes, cellulitis, and cerebral infarction appeared; at 10 years, additional sequelae of cerebrovascular diseases emerged. From 7 years up to diagnosis, tobacco‐related mental disorders and other disorders of electrolyte and fluid balance became characteristic. Unique VaD conditions generally appeared earlier and spanned more time frames than in AD.

Post‐diagnosis, five new VaD conditions emerged, including brain damage from physical disease, pneumonitis, bed sores, as well as other signs and symptoms such as dysphagia, urinary incontinence, senility, head wounds, and fractures (Figure 3). Integrating logistic regression and Bayesian network analyses, comprehensive lists of characteristic conditions for AD before and after diagnosis are detailed in supporting information (Supplementary Results—Subtype lists). Further post‐diagnostic results are in supporting information (Supplementary Results section 4.15).

We examined how comorbidity profiles influence outcomes by analyzing time to death in dementia and control cohorts (Figure S8 and Table S6 in supporting information). We have provided a more detailed overview of related work in supporting information (Supplementary Results section 4.15).

Overall, distinct temporal comorbidity profiles characterized AD and VaD pre‐ and post‐diagnosis. Key associated conditions and potential therapeutic targets were identified via logistic regression and network centrality. Up to 15 years pre‐diagnosis, AD was linked to depressive episodes, epilepsy, osteoporosis, and iron deficiency anemia; VaD to cerebrovascular and peripheral vascular disease, type 1 diabetes, tobacco‐related disorders, arthritis, and overlapping conditions like epilepsy. Within 4 years post‐diagnosis, both subtypes showed bacterial/viral infections, renal failure, fractures, falls, and head injuries. Dementia subtype‐specific lists are in supporting information (Supplementary Results section 4.16).

### 3.5 Results summary

Figure 5 summarizes findings across multi‐layered analyses. Dementia cases showed a substantially higher comorbidity and health‐care contact burden than controls (see section 3.1). Temporal network trajectories indicated that cardio‐metabolic and vascular conditions form an early, highly connected foundation in both AD and VaD, long before diagnosis (see sections 3.3 and 3.4). Logistic regression confirmed that in VaD, this backbone is independently associated with decades pre‐diagnosis, with type 2 diabetes and hypertension persisting across windows and cerebrovascular events strengthening closer to diagnosis (see section 3.2). In the final 0 to 2 years pre‐ and post‐diagnosis, both AD and VaD networks shift toward a shared frailty and complications profile (falls, delirium, dehydration, infections, incontinence, pressure sores, fractures), consistent with escalating functional decline and acute care needs (Figure 3).

### 3.6 Control disease validation

To validate the network‐derived patterns for AD and VaD, we repeated the analysis in a hip fracture population (Figures S9 and S10 in supporting information). While many comorbidities overlapped with those seen in dementia, none were identified as characteristic of hip fractures (Figure 3), supporting the specificity of the patterns observed in AD and VaD.

### 3.7 Cohort demographics

Demographics are shown in Figure S11 in supporting information and ethnicity by subtype in Table S7 in supporting information. ICD‐10 hospital records spanned a median of 16.90 years (interquartile range [IQR] = 10.01 years) for dementia and 13.36 years (IQR = 13.52 years) for controls. Both dementia subtypes had significantly higher PRSs than matched controls (*P *< 0.001), with AD also higher than VaD. Dementia cohorts had more comorbidities than controls (Figure S11d). Of the total AD population, 91% and 92% were either White or British, from the dementia or control cohort, respectively. Equally, the rates of White or British were as high as 90% and 92% for the VaD group of dementia and controls.

## 4 DISCUSSION

We address how comorbidities develop over time, not in isolation. Psychiatric and intestinal disorders develop decades before AD onset.^27^ Cardiovascular comorbidities and type 1 diabetes are linked to both subtypes.^28^ Arthritis is associated with VaD and supports hypotheses around autoimmune contributions to dementia.^29^, ^30^, ^31^ At this stage, we are unable to discern whether these conditions are considered early risk markers or in fact contribute to the disease progression themselves, as analyses featured here were largely statistical in nature identifying correlations and common patterns or themes. As these comorbidities can develop across the lifespan, from early‐, mid‐, and late‐life, a systemic or combined approach may contribute to an increasing instability of disease state, leading to more or less susceptibility to the development of either AD or VaD. Further work factoring in different medications, dosages and adherence would further clarify how well comorbidities are managed and controlled.

Characteristic to AD, from early on, were changes in food and fluid intake, consistently not observed in the VaD cohort. In AD literature, changes to eating and drinking behaviors are frequently associated with patients already diagnosed with AD; however, these changes are not always recognized as preceding diagnosis.^32^ Changes to eating and drinking patterns may also refer to behavior changes, therefore serving as an important potential screening tool or flag for early identification and intervention.^33^ The VaD cohort showed additional metabolic disorders that were characteristic of VaD, more so than AD. In general, type 1 and type 2 diabetes mellitus have both been associated with cognitive dysfunction and dementia.^34^ Poorer glycemic control has been associated with a greater risk of dementia.^35^ Further work with comparison across different electronic health‐care records in both primary and secondary care settings would increase fidelity of these findings.

After diagnosis, respiratory infections and complications were prominent in both AD and VaD. Early identification and management may reduce downstream issues like injuries, falls, and infections.^36^, ^37^ Chronic respiratory infections, pneumonitis, and skin ulcers were characteristic of both cohorts, likely arising from earlier conditions. Our previous work shows that advanced dementia patients increasingly depend on community services.^38^ Targeting these complications can help optimize community care resources and reduce hospital admissions and primary care demand.

Second, we demonstrate potential immediate effects on health care. Complications identified in time‐specific signatures, such as falls and delirium close to diagnosis, have a direct impact on health services and quality of life.^39^ In AD, delirium shows a strong, interwoven relationship with disease progression;^40^ individuals with AD are at higher risk of delirium, creating a cycle of worsening cognitive decline.^37^ A shared pathophysiological basis is likely, with overlapping blood biomarkers and inflammatory processes observed in both conditions.^41^ Early management is therefore critical for patient outcomes and reducing health and social care burden. However, post‐diagnostic or late pre‐diagnostic spikes in delirium may partially reflect hospital coding practices and should be interpreted cautiously.

Finally, we identify comorbidities and intervention windows with longer term implications. Defining diagnosis‐specific time windows enables targeted screening for individuals at elevated risk of AD or VaD. VaD showed greater pre‐diagnostic variation in psychiatric conditions, including depression and substance‐related disorders, with smoking remaining a key modifiable risk.^5^ Earlier intervention may alter disease trajectories. In AD, depressive episodes were more persistent and emerged earlier, consistent with prior studies.^42^, ^43^ Apathy, often under‐coded in hospital data, may further contribute to this relationship.^44^, ^45^

These findings have implications for the design of clinical and therapeutic trials in AD and VaD.^46^ Trial cohorts should include participants with characteristic pre‐dementia comorbidity profiles; otherwise, trials risk under‐representing the heterogeneity of AD and VaD, introducing bias in outcomes and interpretation.^47^

Our results align with recent work.^17^ Despite methodological differences, both studies identified depression and cerebrovascular disease as key features of AD progression. We observed depressive episodes up to 20 years before diagnosis, alongside fluid and food intake issues and falls emerging closer to diagnosis. For VaD, shared pathways included cerebral infarction and cerebrovascular disease, with additional associations observed for type 1 diabetes, arthritis, and intra‐cerebral hemorrhage. Modeling these relationships with temporal specificity enhances clinical interpretability and supports subtype‐stratified analysis.

By adopting a combined temporal approach, we capture how comorbidities interact across disease stages rather than occurring in isolation. Consistent with prior work, cardiovascular disease and type 1 diabetes were closely linked,^28^ while autoimmune conditions such as arthritis, previously associated with VaD,^29^ emerged as part of a broader interacting disease network.

### 4.1 Limitations and future work

#### 4.1.1 Dataset and population

The dataset combines health‐care records with diagnoses reported a median of 2.25 years late, likely under‐representing early‐stage disease. Nevertheless, it provides a suitable resource to examine temporal effects and comorbidity patterns across dementia subtypes.^48^ We prioritized in‐patient hospital data to maximize sample size and capture clinically significant conditions, as hospital admissions often reflect critical points in disease progression and unmanaged or advanced disease. Restricting analyses to in‐patient records, excluding primary care and self‐reported diagnoses, likely reflects a more severe population; however, these individuals disproportionately drive health‐care use and service burden over the disease course.

As with all electronic health‐care record studies, diagnostic timing and “dump” events—when multiple codes are recorded on a single date—represent key limitations, reflecting episodic engagement with records. Important clinical details may reside in free‐text notes not available in UK Biobank. Future integration of such data, subject to ethical approval, could improve diagnostic precision. We assumed diagnoses to be permanent once recorded, as acute versus chronic status cannot be reliably distinguished; this may inflate comorbidity burden. Future work will address this by weighting acute and chronic conditions differently (Supplementary Methods Section 4.9). The lower comorbidity burden and mortality observed in population controls reflects known limitations of in‐patient datasets, in which individuals without dementia have fewer repeat admissions, yielding an apparently healthier control group.

Most of the cohort identified as British (Figure S11), reflecting limited ethnic diversity in the UK Biobank. This should be considered when interpreting generalizability, particularly given known inequalities in dementia diagnosis, care, and hospitalization among minority groups.^49^ Sampling bias was not imputed, as the aim was to characterize dementia populations captured within in‐patient care.

#### 4.1.2 Technical limitations

Standard UK Biobank PRSs for AD were examined across groups,^22^, ^23^ but were not used for individual‐level matching; future studies should incorporate this. Replication in independent longitudinal cohorts is also required to assess reproducibility.

## AUTHOR CONTRIBUTIONS

C.W.: conceptualization, methodology, software, formal analysis, investigation, data curation, validation, visualization, writing—original draft, review and editing; A.F.: software, review and editing; A.K.S.: software, review and editing; V.M.: writing—original draft, review and editing; B.S.: writing—original draft, review and editing; C.S.: review and editing; M.R.: review and editing; R.N.: conceptualization, methodology, writing—original draft, review and editing, supervision; P.B.: conceptualization, methodology, writing—original draft, review and editing, supervision, funding acquisition.

## FUNDING INFORMATION

This study is funded by the UK Dementia Research Institute (UK DRI) Care Research and Technology Centre funded by the Medical Research Council (MRC; UKDRI‐7002), Alzheimer's Research UK, Alzheimer's Society (grant number: UKDRI‐7002), and the UKRI Engineering and Physical Sciences Research Council (EPSRC) Resilient Project (grant number: EP/W031892/1). Infrastructure support for this research was provided by the NIHR Imperial Biomedical Research Centre (BRC; NIHR203323) and the UKRI Medical Research Council (MRC). P.B. is funded by Great Ormond Street Hospital and the Royal Academy of Engineering (RCSRF2324‐18‐69). C.S. and A.K.S are supported by the UK Dementia Research Institute (UK DRI‐5209) and a UKRI Future Leaders Fellowship (MR/X032892/1). C.S. also receives personal support from the Edmond J. Safra Foundation.

## CONSENT STATEMENT

All human subjects provided their fully informed consent.

## CONFLICT OF INTEREST STATEMENT

All authors declare no financial or non‐financial competing interests. Author disclosures are available in the supporting information.

## Supporting information



Supporting Information

Supporting Information

## Data Availability

The data that support the findings of this study are available from the UK Biobank, found here: https://www.ukbiobank.ac.uk. The cohort populations that we have identified will be also made available from UK Biobank, upon publication. The code used in this study will be made available by the corresponding author on reasonable request.
