# Supplementary material for: Temporal comorbidity patterns in Alzheimer's disease and vascular dementia: A population‐based observational study in UK Biobank
Source: Alzheimers Dement (Amst). 2026 Feb 10;18(1):e70265. doi: 10.1002/dad2.70265 (PMC12887687; doi:10.1002/dad2.70265)
Supplement: Supplementary file 1 — Supporting Information [file DAD2-18-e70265-s002.pdf]

Supplementary Methods

4.1 Cohort selection

Within the UK Biobank, there are pre-existing datasets that define a population. An example of this is the 'All Cause Dementia' dataset. However, this data included participants who self-reported their dementia diagnosis. We emphasised formal diagnosis and focused on in-patient records; therefore, we defined our cohort from ICD-10 coding instead.

4.2 Categorising Comorbidities Using ICD-10 Codes, Blocks and Chapters

The ICD-10 coding system is a hierarchical system for categorising diseases, comorbidities and complications of disease. The top level are Chapters (1-22), followed by blocks (A00-Z99; approximately 221), which further classify diseases within each Chapter. Following blocks, there are Categories and Sub-categories. The UK Biobank dataset provides the individual ICD-10 codes for the first instance of each diagnosis per participant; these individual codes were the primary source for this analysis. Counts of each individual diagnosis were calculated for dementia and control groups. We further categorised each individual code into its corresponding block and chapter using the World Health Organisation ICD-10 Application Programme Interface (API), 2019 release[20].

4.3 Diagnosis grouping

To accurately capture the clinical diagnosis of participants with more than one dementia diagnosis in their records, we established a standardised and reproducible approach, guided by a group of clinical experts within the Surrey and Borders Partnership NHS Foundation Trust. The clinical team comprised of Consultant Old Age Psychiatrists and dementia clinical experts mapped each participant to an 'Overall Diagnosis' from multiple ICD-10 codes. The criteria were as follows: AD and VD were considered 'primary' diagnoses. Mild Cognitive Impairment, and Unspecified Dementia were considered as 'secondary' diagnoses. All remaining diagnoses were collectively named 'Other', including Lewy Body Dementia, Dementia in Parkinson's and Dementia in Huntington's were considered 'secondary' diagnoses.

From the lists of multiple diagnosis per individual, we created a pipeline that established an 'Overall Diagnosis' for each participant, made up of: AD, VD, Mild Cognitive Impairment, Unspecified Dementia and Other. We excluded all diagnoses except AD and VD based on clinical uncertainty from data coding. Therefore, counts of each primary diagnosis were calculated for each individual, and the majority diagnosis was mapped as 'Overall Diagnosis' for each participant. AD was assumed in all instances where: AD was the most prevalent diagnosis and equal counts of either AD or VD. VD was assumed if VD was the

most prevalent diagnosis. Overall, this resulted in a cohort of participants who had a diagnosis of either AD (n=3867) or VD (n=1481), a total cohort of 5,348 participants with AD or VD. A schematic of this process is detailed in Figure 1.

4.4 Diagnosis date

The first date of each dementia diagnosis was used as the dementia onset date. This was also true for participants with multiple diagnoses in their records. This date was then assigned to the matched control as a 'placeholder diagnosis flag' date.

4.5 Control Cohort

After identifying 5,348 dementia cases, controls were matched by sex, birth year, and death date using a fixed random seed and validation to control demographic and survival differences. These variables were selected as primary matching criteria to control for demographic factors that could influence dementia risk or survival outcomes. Dementia participants were grouped by sex and age at diagnosis.

4.6 Control cohort selection

Dementia cases were excluded from the UK Biobank, leaving 490,467 potential controls. Controls matched dementia cases by sex and age, had no dementia diagnosis, and complete inpatient data. Each control was used once and had to be alive at or after the matched dementia case's diagnosis or death date. From up to three potential matches per case, one control was randomly selected (fixed seed) to create a 1:1 matched cohort of 10,508 participants. As a verification for control groups, we compared the Polygenic Risk Scores (PRS) of 'Standard PRS for AD' between dementia and control groups. An overall comparison between groups was performed using Mann-Whitney U testing, corrected for multiple comparisons across all comparisons using False Discovery Rate Benjamini-Hochberg.

For control participants, a placeholder index date was assigned to establish a common temporal reference point for aligning longitudinal trajectories, as controls do not have a dementia diagnosis by definition. This index date enabled consistent comparison of pre- and post-event time windows across groups. All control participants were drawn from in-patient hospital records; therefore, each had documented contact with secondary care, reflected in their medical histories. While the use of a placeholder index date is an acknowledged methodological limitation, it represents a pragmatic and widely adopted approach in longitudinal observational studies using routinely collected healthcare data, where a true clinical event date is unavailable.

4.7 Rationale for Temporal Windows in Comorbidity Analysis

The categorisation of comorbidities into discrete pre-diagnosis time frames and overlapping post-diagnosis windows was a deliberate methodological decision informed by the nature of inpatient hospital coding and extensive sensitivity testing. Pre-diagnosis time frames were defined as non-overlapping categorical windows (e.g., 20+ years, 15–20 years, 10–15 years, etc.) to reflect phases where diagnostic coding is largely independent of dementia recognition. During this phase, recorded comorbidities are more likely to represent clinically meaningful health events occurring in real time rather than being influenced by the dementia diagnostic process itself. Non-overlapping windows therefore tend to maintain temporal specificity, similar to any other individual from the general population and allow clearer differentiation of when conditions emerge relative to dementia onset. Small differences (e.g., 14 vs 16 years before diagnosis) are intentionally absorbed within broader windows to prioritise clinical relevance and robustness over precision that is unlikely to be reliable in routinely collected hospital data. With more granular data, we would look to refine these windows, alas this data set does not allow for this currently.

In contrast, post-diagnosis coding reflects a fundamentally different clinical and administrative context. Following a dementia diagnosis, inpatient records increasingly capture an accumulating comorbidity profile, driven by greater healthcare contact, diagnostic clustering, and repeated documentation of existing conditions. In this context, non-overlapping post-diagnosis windows risk fragmenting a cumulative process and under representing the evolving burden of comorbidity.

We therefore adopted overlapping post-diagnosis windows (e.g., 0–2, 0–4, 0–6, 0–8, and 0–10 years after diagnosis) to model the progressive accumulation of comorbidities over time rather than discrete, mutually exclusive periods. This approach enables examination of how the influence and centrality of conditions evolve as dementia progresses, which is more consistent with clinical trajectories observed after diagnosis.

4.8 Analysis overview

The analyses were intentionally designed as a multi-layered, complementary framework to examine comorbidities of AD and VD from different analytic perspectives, rather than relying on a single statistical approach. The order of analyses reflects increasing analytic specificity and inferential strength.

First, Mann–Whitney U tests were conducted as an exploratory, group-level comparison between dementia cases and controls to establish whether overall differences in comorbidity burden were present. This step served as an initial screening to justify more granular analysis.

Coding of each diagnosis, per individual, were stratified into time frames or windows. Pre- and post-diagnosis time frames

were intentionally structured differently to reflect known differences in inpatient coding behaviour before and after dementia diagnosis, with non-overlapping windows used to preserve temporal specificity pre-diagnosis and overlapping windows used post-diagnosis to capture cumulative comorbidities.

Second, sequence analysis followed by network analysis was performed within each time frame and diagnostic group (AD vs controls; VD vs controls). These analyses were designed to characterise the structural organisation, temporal ordering, and centrality of comorbid conditions within the broader comorbidity landscape. Network centrality identifies conditions that are highly connected or influential within the system but does not assess their independent contribution to disease outcomes.

Finally, logistic regression models were applied within the same stratified time frames and diagnostic groups to quantify the predictive association between individual comorbidities and dementia outcomes (AD or VD vs controls). These models estimate effect sizes and directionality but do not capture interdependencies between conditions.

As an outcome measure, mortality in each of the dementia and control groups is important to consider to grasp an overall picture of the impact of progression in these populations. We applied Kaplan-Meier Survival analysis for each sub-type vs controls. We converted survival probabilities into the equivalent time in months that each sub-type of dementia were ahead of the control population.

4.9 Network analysis of comorbidities across time points

To account for the impact of comorbidities across an individual's health landscape leading up to dementia diagnosis, we conducted an undirected network analysis[49]. The network analysis was performed for each time frame and each dementia sub-type. Undirected graphical models are also known as Markov Random Fields. Markov Random Fields are similar to Bayesian networks but differ in that they use undirected edges to represent non-causal dependencies. These probabilistic models can represent correlations and conditional dependencies between variables in complex systems. In the network analysis, and for each time frame, at least 100 different individuals were included for both dementia and control cohorts. This was conducted using only the conditions most significantly (p-value <0.05) associated with a dementia diagnosis, as determined by the two-sided Mann-Whitney U test.

A minimum support threshold of 50 was used for ICD-10 individual diagnostic codes. However, this was increased to 100 for analysis of ICD-10 blocks. These thresholds were chosen to ensure the analysis focused on clinically insightful patterns while reducing noise from infrequent combinations. Centrality measures were calculated for the conditions (i.e. nodes) within each network, specific to time frame and dementia sub-type, and replicated for matched controls. Each diagnosis, per individual, was categorised into a time frame relative to the date of first diagnosis of dementia and split to AD or VD. The matched

controls were assigned to the dementia sub-type in which their corresponding dementia participant was. Once the networks for each time frame and subtype were established, the networks were compared across the time frames. Conditions unique to the first network (20+ years before diagnosis) were identified across the dementia and control groups and marked as re-occurring when identified across consecutive time frames. Therefore, newly diagnosed conditions were identified at each time frame per dementia sub-type. The same analysis was repeated for ICD-10 blocks, with a minimum support of 100.

An assumption was made, such that from diagnosis of each comorbidity, that diagnosis remained with that individual. If there was a repeat diagnosis, this was counted in addition to the previous diagnosis. Clinically, not all comorbidities are alike; some are diagnosed, managed and resolved and some are unresolved or become chronic, however, for the purposes of this analysis, all conditions were considered to remain present following initial diagnosis and were not removed from individual records. This will reduce the risk of underestimating the effect of comorbidities. This approach also reflects the reality of conditions, especially in older populations, which are often progressive, chronic or complex with long-lasting effects.

4.10 Control disease selection - Hip fracture

To distinguish dementia-specific comorbidity patterns from general ageing effects, we analysed a hip fracture cohort (ICD-10 '$S72$'), a common non-neurological condition in older adults sharing ageing risk factors. Hip fracture cases were matched 1:1 with controls without hip fracture, excluding individuals in dementia and control cohorts. All groups were matched on sex and age at diagnosis.

During study design, clinicians were consulted to address a related conceptual issue: whether observed associations for the control disease reflected normative ageing processes or mechanisms specific to dementia. Several chronic conditions prevalent in older populations, including cardiovascular and metabolic diseases, are also independently associated with dementia risk. Matching controls with a high comorbidity burden or frequent hospitalisation would therefore risk over-adjustment and detection of dementia-specific associations, potentially undermining the primary objective of the study.

To address this, a sensitivity analysis was conducted using hip fracture patients as an alternative control group. Hip fracture represents an acute, age-related event that necessitates hospitalisation but is not intrinsically part of the dementia disease process. This comparison allowed us to assess whether observed patterns were driven primarily by general hospitalisation or ageing rather than dementia-related mechanisms. Hip fracture controls were intentionally restricted to this sensitivity analysis to avoid conflating distinct control definitions within the main analytical framework. This strengthened the robustness of the findings; conditions unique to the AD and VD cohorts, not found in the hip fracture cohort was more likely to be a genuine reflection of the population as opposed to conditions more likely at specific time windows throughout life, as we age.

Supplementary Results

4.11 Control cohort and control disease validation (Hip fracture)

Polygenic risk scores across dementia and control groups revealed statistically significant differences, across and within dementia sub-types. Standard PRS for AD was significantly different between dementia and controls (corrected *p-value* <0.001). There was also a significant difference within AD (corrected *p-value* <0.001) and VD (corrected *p-value* <0.001) groups.

To verify the findings that we had observed with AD and VD, we repeated the analysis using a control disease. This was identified as hip fractures, denoted by code '$S72$' in ICD-10 coding. Supplementary Figure S9 shows the top 20 conditions that were identified as significantly associated with the cohort diagnosed with hip fractures, when compared with a population without hip fractures. Confirming our findings, these lists did not have any conditions in common with characteristic conditions of the AD or VD population. In addition, we analysed the prevalence of the conditions identified in Figure 2, in the cohort of people with hip fractures. Supplementary Figure S10 details the proportion of people with hip fractures, that were diagnosed with the top 20 most significant conditions associated with a dementia diagnosis of either AD or VD, as seen in the dementia cohorts (Figure 2).

4.12 Machine learning model selection

Several models were used to establish the predictive ability of conditions diagnosed in each time frame before dementia diagnosis, separately for AD and VD (Supplementary Table S2 and S3). Logistic regression with L1 regularisation was selected as the most appropriate model. This was the best performing model for VD overall, and there were small differences between this model and other models for AUC in the AD cohort. In earlier time frames, the models show lower AUC performance, reflecting that at distant horizons, comorbidity patterns are not yet strongly predictive of AD or VD risk. Early window time frames showed weak predictive potential however, this was only used as an indication, as opposed to the primary outcome of the analysis (Supplementary Table S4). In addition, VD cohorts have shown more distinct patterns of conditions over time, particularly leading up to diagnosis. Therefore, as we did not aim to predict if people will be diagnosed with either AD or VD, we only set out to determine the predictive ability of conditions with respect to dementia sub-type; performance was not prioritised for model selection.

4.13 Logistic regression analysis breakdown

Here, we provide a more detailed description of the the regression analysis for AD and VD cohorts. Although there were fluctuations in odds ratio values across the different cohorts, some trends can be observed for each subtype. For AD subtype, odds ratio measures for conditions such as delirium and extrapyramidal and movement disorders increased as the time frames got closer to diagnosis (Figure 4a). However, conditions such as epilepsy showed a reduction in the odds ratio across the time

frames. For VD, intracerebral haemorrhage showed an increase in odds ratio across the time frames, as we get closer to the diagnosis. Reduction in odds ratios for type 2 diabetes were observed in the VD cohort (Figure 4b).

In the AD cohort, 10–7 years before diagnosis, symptoms and signs concerning food and fluid intake (Odds Ratio [OR]: 2.28, 95% CI: 1.42–3.68, $p = 0.001$), extrapyramidal and movement disorders (OR: 4.00, 95% CI: 1.41–11.38, $p = 0.009$), chest pain (OR: 1.39, 95% CI: 1.14–1.68, $p = 0.001$), and symptoms involving cognitive functions and awareness (OR: 2.38, 95% CI: 1.18–4.80, $p = 0.016$) were characteristic (Figure 4a).

From 7–5 years before diagnosis, continued associations were observed for food and fluid intake symptoms (OR: 1.92, 95% CI: 1.28–2.86, $p = 0.001$), extrapyramidal and movement disorders (OR: 3.35, 95% CI: 1.43–7.87, $p = 0.005$), and bipolar affective disorder (OR: 3.87, 95% CI: 1.56–9.63, $p = 0.004$). Between 5–2 years before diagnosis, associations intensified for cognitive symptoms and awareness (OR: 3.68, 95% CI: 2.51–5.39, $p < 0.001$), delirium (OR: 3.57, 95% CI: 1.83–6.94, $p < 0.001$), extrapyramidal and movement disorders (OR: 4.26, 95% CI: 2.00–9.06, $p < 0.001$), and food and fluid intake symptoms (OR: 2.46, 95% CI: 1.77–3.42, $p < 0.001$). Depressive episodes were also significantly associated during this interval (OR: 1.39, 95% CI: 1.05–1.84, $p = 0.024$).

Within the final two years prior to diagnosis, the strongest associations were observed for cognitive symptoms and awareness (OR: 5.38, 95% CI: 3.91–7.40, $p < 0.001$), delirium (OR: 4.26, 95% CI: 2.54–7.15, $p < 0.001$), and extrapyramidal and movement disorders (OR: 4.49, 95% CI: 2.27–8.86, $p < 0.001$). Food and fluid intake symptoms remained strongly associated up to diagnosis (OR: 2.36, 95% CI: 1.78–3.15, $p < 0.001$), alongside emerging associations with unspecified diabetes mellitus (OR: 1.85, 95% CI: 1.12–3.04, $p = 0.016$) and falls (OR: 1.56, 95% CI: 1.08–2.26, $p = 0.017$) (Figure 4a).

For the VD cohort, from 20–15 years before diagnosis, type 2 diabetes mellitus without complications was strongly associated with a VD diagnosis (OR: 2.90, 95% CI: 1.31–6.39, $p = 0.008$) (Figure 4b). This association remained consistent and sustained across all subsequent time frames up to diagnosis. From 15–10 years before diagnosis, strong associations emerged for cerebrovascular disease (OR: 5.44, 95% CI: 1.49–19.86, $p = 0.010$), epilepsy (OR: 7.16, 95% CI: 1.99–25.85, $p = 0.003$), and stroke not specified as haemorrhage or infarction (OR: 4.83, 95% CI: 1.52–15.31, $p = 0.008$). During this period, hypertensive diseases (OR: 1.42, 95% CI: 1.05–1.94, $p = 0.025$) also showed significant associations (Figure 4b).

Between 10–7 years before diagnosis, stroke remained strongly associated (OR: 5.85, 95% CI: 1.79–19.14, $p = 0.004$), alongside epilepsy (OR: 4.63, 95% CI: 1.65–12.98, $p = 0.004$). Hypertensive diseases continued to show a stable association (OR: 1.41, 95% CI: 1.08–1.83, $p = 0.011$). A significant association was also observed for personal history of circulatory system disease (OR: 1.39, 95% CI: 1.07–1.82, $p = 0.016$).

From 7–5 years before diagnosis, intracerebral haemorrhage emerged as a strong predictor of VD (OR: 3.42, 95% CI: 1.07–10.90, $p = 0.038$). Additional associations were observed for epilepsy (OR: 2.50, 95% CI: 1.08–5.79, $p = 0.033$), depressive episodes (OR: 1.78, 95% CI: 1.01–3.13, $p = 0.046$), and hypertensive diseases (OR: 1.40, 95% CI: 1.10–1.77, $p = 0.007$). Within 5–2 years before diagnosis, associations intensified for intracerebral haemorrhage (OR: 5.17, 95% CI: 1.99–13.42, $p = 0.001$), cerebral infarction (OR: 2.45, 95% CI: 1.37–4.39, $p = 0.003$), and transient cerebral ischaemic attack (OR: 3.40, 95% CI: 1.31–8.84, $p = 0.012$). Cognitive symptoms and awareness became strongly associated during this period (OR: 4.12, 95% CI: 1.95–8.69, $p < 0.001$). Psychiatric comorbidities including recurrent depressive disorder (OR: 5.57, 95% CI: 1.32–23.58, $p = 0.020$) and schizophrenia (OR: 6.03, 95% CI: 1.56–23.35, $p = 0.009$) were also significantly associated.

In the final two years prior to diagnosis, strong associations were observed for delirium (OR: 3.80, 95% CI: 1.39–10.38, $p = 0.009$), and bipolar affective disorder (OR: 5.84, 95% CI: 1.74–19.63, $p = 0.004$). Mental and behavioural disorders due to tobacco use (OR: 1.81, 95% CI: 1.02–3.22, $p = 0.043$), depressive episodes (OR: 2.07, 95% CI: 1.21–3.54, $p = 0.008$) and transient cerebral ischaemic attack (OR: 2.57, 95% CI: 1.07–6.18, $p = 0.036$) remained significantly associated up to diagnosis (Figure 4b).

4.14 Undirected network analysis of additional complications and symptoms of Alzheimer's Disease

Symptoms range from headache to symptoms concerning food and fluid intake. From 15 to 7 years up to diagnosis of AD, symptoms such as retention of urine were the only new, unique symptoms for the AD cohort. Up to 5 years before AD diagnosis, invertebral disc disorder was the only new, unique condition associated with the AD cohort and signs of loss of cognitive functions and awareness at this time (Figure 3). Up to 2 years before diagnosis, falls and bacterial agents as cause of diseases emerged, followed by falls from slipping/tripping/stumbling in the remaining 2 years before AD (Figure 3). Symptoms identified closest to AD include nervous system and musculoskeletal symptoms as well as symptoms related to abnormality of gait. At this time frame, cerebrovascular diseases and delirium were also characteristic of the AD cohort, compared to controls; however, a similar pattern was observed with VD. Symptoms involving concern of fluid and food intake as well as depressive episodes were seen to re-occur over at least five different time frames (Figure 3). Most other conditions were then shared amongst the age- and sex-matched control cohorts.

4.15 Outcomes post-diagnosis of Alzheimer's Disease and Vascular Dementia

We examined how comorbidity profiles influence outcomes by analysing time to death in dementia and control cohorts (Supplementary Figure S8). The survival analysis incorporated covariates of age and sex, as well as the different dementia subtypes

according to 'Overall Diagnosis'. HR for each of the 'Overall Diagnosis' groups were calculated, and the time to death at survival probability intervals was calculated for the control cohort. For AD and VD, the time in months that the dementia cohort was ahead of the controls was calculated; i.e. an individual with dementia would die X months sooner than a control individual of the same sex and age at the time of the dementia diagnosis. Survival analysis showed a faster decline in those with dementia compared to age- and sex-matched controls (Figure S8).

Analysis was limited to 67 months post-diagnosis. Survival lag quantified how much earlier dementia groups reached equivalent survival probabilities (Figure S8); results beyond 0.5 probability are in Supplementary Table S6. From diagnosis to six months post-diagnosis, controls survived 11 months longer than AD and 14 months longer than VD patients. This trend persisted, with VD declining faster than AD.

4.16 Sub-type specific lists

AD:'Other symptoms and signs concerning food and fluid intake', 'Other symptoms and signs involving cognitive functions and awareness', 'Depressive episode', 'Intervertebral disc disorder', 'Epilepsy', 'Delirium', 'Unspecified fall', 'Other symptoms and signs involving the nervous and musculoskeletal systems'. VD: 'Cerebral infarction', 'Care involving use of rehabilitation procedure', 'Cerebrovascular disease', 'Arthritis', 'Epilepsy', 'Hypotension', 'Other and unspecified symptoms and signs involving cognitive functions and awareness', 'Mental and behavioural disorders due to use of tobacco', ''Mental and behavioural disorders due to use of alcohol', 'Delirium', 'Depressive episode', 'Falls', 'Type 1 diabetes mellitus without complications', 'Other abnormalities of gait and mobility'.

Supplementary Tables

Table S1: Summary of standardised World Health Organisation (WHO) International Statistical Classification of Diseases and Related Health Problems 10th Revision (ICD-10), version 2019. Comorbidities and health conditions are coded using this standardised system across UK Biobank records. This forms a hierarchical structure where there are a total of 22 chapters. Within each chapter are a range of codes, labelled 'Blocks'. Within these are further detailed codes referring to 'Categories'; individual health conditions and types. For simplicity, the analysis in this work uses mainly three/four code ICD-10 conditions, categories and within category codes. (AD: Alzheimer's Disease, VD: Vascular Dementia).

ICD-10 Chapter	Chapter Title	Code Range	Diagnostic Blocks (Summary)
I	Certain infectious and parasitic diseases	A00–B99	Intestinal infections; tuberculosis; zoonotic bacterial diseases; other bacterial and viral diseases; sexually transmitted infections; mycoses; protozoal and helminthic diseases; sequelae of infectious diseases
II	Neoplasms	C00–D48	Malignant neoplasms by anatomical site; melanoma; lymphoid and haematopoietic malignancies; in situ neoplasms; benign neoplasms; neoplasms of uncertain or unknown behaviour
III	Diseases of the blood and blood-forming organs and certain disorders involving the immune mechanism	D50–D89	Nutritional, haemolytic, aplastic and other anaemias; coagulation defects; immune mechanism disorders
IV	Endocrine, nutritional and metabolic diseases	E00–E90	Thyroid disorders; diabetes mellitus; disorders of glucose regulation; endocrine gland disorders; malnutrition; obesity; metabolic disorders
V	Mental and behavioural disorders	F00–F99	Organic mental disorders; substance use disorders; schizophrenia and delusional disorders; mood disorders; anxiety and stress-related disorders; eating disorders; personality disorders; developmental and childhood-onset disorders
VI	Diseases of the nervous system	G00–G99	Inflammatory, degenerative, demyelinating and other disorders of the nervous system
VII	Diseases of the eye and adnexa	H00–H59	Disorders of eyelids, conjunctiva, lens, retina, and visual pathways
VIII	Diseases of the ear and mastoid process	H60–H95	External, middle and inner ear diseases; hearing and balance disorders
IX	Diseases of the circulatory system	I00–I99	Hypertensive diseases; ischaemic heart disease; cerebrovascular disease; venous and lymphatic disorders
X	Diseases of the respiratory system	J00–J99	Upper and lower respiratory infections; chronic respiratory diseases; lung disorders
XI	Diseases of the digestive system	K00–K93	Disorders of the oral cavity; gastrointestinal, hepatic, biliary and pancreatic diseases
XII	Diseases of the skin and subcutaneous tissue	L00–L99	Infections of the skin; dermatitis; inflammatory and degenerative skin conditions
XIII	Diseases of the musculoskeletal system and connective tissue	M00–M99	Arthropathies; systemic connective tissue disorders; musculoskeletal pain and degenerative conditions
XIV	Diseases of the genitourinary system	N00–N99	Renal disease; urinary tract disorders; male and female genital conditions
XV	Pregnancy, childbirth and the puerperium	O00–O99	Pregnancy-related conditions; obstetric complications; maternal care
XVI	Certain conditions originating in the perinatal period	P00–P96	Conditions affecting newborns related to pregnancy, labour and delivery
XVII	Congenital malformations, deformations and chromosomal abnormalities	Q00–Q99	Structural anomalies; genetic and chromosomal disorders
XVIII	Symptoms, signs and abnormal clinical and laboratory findings	R00–R99	Non-specific symptoms; abnormal findings without definitive diagnosis
XIX	Injury, poisoning and certain other consequences of external causes	S00–T98	Traumatic injuries; burns; poisonings; complications of medical care
XX	External causes of morbidity and mortality	V01–Y98	Accidents; intentional self-harm; assault; adverse events
XXI	Factors influencing health status and contact with health services	Z00–Z99	Health service encounters; social circumstances; follow-up and screening
XXII	Codes for special purposes	U00–U99	Emergency use codes; antimicrobial resistance; provisional classifications

Table S2: Model performance metrics for classification machine learning models for Alzheimer's Disease. Comorbidity diagnosis as inputs, confounding for age at diagnosis of AD and sex. (AD: Alzheimer's Disease, AUC: Area Under the Receiver Operating Characteristic Curve).

Time Frame	Model Type	Sensitivity	Specificity	Accuracy	Precision	F1-Score	AUC
20+ years before AD	Logistic Regression (L1)	0.420	0.568	0.497	0.474	0.446	0.471
20+ years before AD	Logistic Regression (L2)	0.477	0.505	0.492	0.472	0.475	0.469
20+ years before AD	Random Forest	0.420	0.568	0.497	0.474	0.446	0.487
20+ years before AD	XGBoost	0.466	0.505	0.486	0.466	0.466	0.489
20-15 years before AD	Logistic Regression (L1)	0.619	0.559	0.592	0.628	0.624	0.590
20-15 years before AD	Logistic Regression (L2)	1.000	0.000	0.546	0.546	0.706	0.540
20-15 years before AD	Random Forest	0.615	0.473	0.551	0.584	0.599	0.538
20-15 years before AD	XGBoost	0.624	0.441	0.541	0.573	0.597	0.550
15-10 years before AD	Logistic Regression (L1)	0.626	0.477	0.552	0.552	0.586	0.571
15-10 years before AD	Logistic Regression (L2)	1.000	0.000	0.507	0.507	0.673	0.526
15-10 years before AD	Random Forest	0.571	0.438	0.506	0.511	0.539	0.519
15-10 years before AD	XGBoost	0.603	0.462	0.534	0.535	0.567	0.544
10-7 years before AD	Logistic Regression (L1)	0.513	0.552	0.532	0.540	0.526	0.564
10-7 years before AD	Logistic Regression (L2)	0.877	0.167	0.527	0.520	0.653	0.532
10-7 years before AD	Random Forest	0.565	0.504	0.535	0.539	0.552	0.557
10-7 years before AD	XGBoost	0.507	0.576	0.541	0.551	0.528	0.553
7-5 years before AD	Logistic Regression (L1)	0.489	0.614	0.551	0.566	0.525	0.576
7-5 years before AD	Logistic Regression (L2)	0.602	0.406	0.505	0.511	0.553	0.496
7-5 years before AD	Random Forest	0.543	0.555	0.549	0.557	0.550	0.574
7-5 years before AD	XGBoost	0.462	0.673	0.566	0.593	0.520	0.589
5-2 years before AD	Logistic Regression (L1)	0.457	0.696	0.575	0.608	0.522	0.594
5-2 years before AD	Logistic Regression (L2)	0.535	0.481	0.508	0.514	0.524	0.519
5-2 years before AD	Random Forest	0.541	0.612	0.576	0.589	0.564	0.594
5-2 years before AD	XGBoost	0.426	0.724	0.573	0.614	0.503	0.599
2 years before AD	Logistic Regression (L1)	0.536	0.756	0.649	0.674	0.597	0.678
2 years before AD	Logistic Regression (L2)	0.633	0.406	0.516	0.501	0.559	0.527
2 years before AD	Random Forest	0.572	0.699	0.637	0.641	0.604	0.680
2 years before AD	XGBoost	0.500	0.799	0.654	0.700	0.583	0.693

Table S3: Model performance metrics for classification machine learning models for Vascular Dementia. Comorbidity diagnosis as inputs, confounding for age at diagnosis of Vascular Dementia and sex. (VD: Vascular Dementia, AUC: Area Under the Receiver Operating Characteristic Curve)

Time Frame	Model Type	Sensitivity	Specificity	Accuracy	Precision	F1-Score	AUC
20+ years before VD	Logistic Regression (L1)	0.929	0.051	0.506	0.513	0.661	0.485
20+ years before VD	Logistic Regression (L2)	1.000	0.000	0.519	0.519	0.683	0.372
20+ years before VD	Random Forest	0.762	0.179	0.481	0.500	0.604	0.520
20+ years before VD	XGBoost	0.833	0.308	0.580	0.565	0.673	0.582
20-15 years before VD	Logistic Regression (L1)	0.810	0.157	0.549	0.590	0.683	0.562
20-15 years before VD	Logistic Regression (L2)	1.000	0.000	0.600	0.600	0.750	0.480
20-15 years before VD	Random Forest	0.638	0.314	0.509	0.583	0.609	0.517
20-15 years before AD	XGBoost	0.752	0.257	0.554	0.603	0.669	0.518
15-10 years before VD	Logistic Regression (L1)	0.766	0.453	0.633	0.655	0.706	0.685
15-10 years before VD	Logistic Regression (L2)	1.000	0.000	0.575	0.575	0.730	0.540
15-10 years before VD	Random Forest	0.718	0.496	0.624	0.659	0.687	0.669
15-10 years before VD	XGBoost	0.745	0.496	0.639	0.667	0.704	0.706
10-7 years before VD	Logistic Regression (L1)	0.618	0.533	0.582	0.650	0.634	0.647
10-7 years before VD	Logistic Regression (L2)	0.651	0.562	0.614	0.677	0.664	0.654
10-7 years before VD	Random Forest	0.668	0.509	0.602	0.657	0.663	0.612
10-7 years before VD	XGBoost	0.643	0.550	0.604	0.668	0.655	0.652
7-5 years before VD	Logistic Regression (L1)	0.631	0.676	0.652	0.700	0.664	0.693
7-5 years before VD	Logistic Regression (L2)	0.963	0.054	0.549	0.549	0.699	0.512
7-5 years before VD	Random Forest	0.689	0.525	0.614	0.634	0.660	0.654
7-5 years before VD	XGBoost	0.623	0.627	0.625	0.667	0.644	0.685
5-2 years before VD	Logistic Regression (L1)	0.626	0.717	0.668	0.725	0.672	0.713
5-2 years before VD	Logistic Regression (L2)	0.619	0.683	0.648	0.698	0.656	0.683
5-2 years before VD	Random Forest	0.678	0.591	0.638	0.663	0.670	0.694
5-2 years before VD	XGBoost	0.648	0.709	0.676	0.725	0.685	0.716
2 years before VD	Logistic Regression (L1)	0.701	0.818	0.753	0.829	0.760	0.815
2 years before VD	Logistic Regression (L2)	0.617	0.809	0.702	0.803	0.698	0.793
2 years before VD	Random Forest	0.735	0.809	0.768	0.830	0.779	0.850
2 years before VD	XGBoost	0.688	0.873	0.770	0.872	0.769	0.840

Table S4: Significantly associated comorbidities with Alzheimer's Disease cohort from logistic regression model. Significance here refers to p-value <0.05. Conditions are displayed with corresponding odds ratios and 95% confidence intervals. Conditions are displayed in alphabetical order.

Timeframe	Condition	Odds Ratio	CI Lower	CI Upper	P-Value
20+ years before	Hypothyroidism	5.104	1.456	17.895	0.011
20+ years before	Peripheral vascular disease	0.198	0.049	0.798	0.023
20-15 years before	Chest pain	1.493	1.041	2.141	0.029
20-15 years before	Disorder of urinary system	1.810	1.082	3.027	0.024
20-15 years before	Epilepsy	16.500	5.869	46.387	0.000
20-15 years before	Iron deficiency anaemia	8.633	4.135	18.026	0.000
20-15 years before	Peripheral vascular disease	0.198	0.073	0.537	0.001
15-10 years before	Abnormal finding of blood chemistry	0.403	0.202	0.806	0.010
15-10 years before	Angina pectoris	1.366	1.006	1.855	0.046
15-10 years before	Bipolar affective disorder	4.364	1.648	11.556	0.003
15-10 years before	Chest pain	1.305	1.025	1.661	0.031
15-10 years before	Disorder of urinary system	1.582	1.142	2.191	0.006
15-10 years before	Epilepsy	2.465	1.154	5.265	0.020
15-10 years before	Fracture of shoulder girdle, part unspecified	0.406	0.166	0.992	0.048
15-10 years before	Observation for suspected disease or condition	1.694	1.081	2.657	0.022
15-10 years before	Problem related to lifestyle	1.578	1.099	2.266	0.014
15-10 years before	Type 1 diabetes mellitus without complications	2.209	1.159	4.213	0.016
15-10 years before	Type 2 diabetes mellitus without complications	1.411	1.046	1.904	0.024
15-10 years before	Unknown and unspecified causes of morbidity	1.393	1.008	1.925	0.044
10-7 years before	Chest pain	1.385	1.141	1.680	0.001
10-7 years before	Extrapyramidal and movement disorders	4.004	1.409	11.378	0.009
10-7 years before	Fracture of other and unspecified parts of lumbar spine and pelvis	0.370	0.147	0.930	0.034
10-7 years before	Other and unspecified symptoms and signs involving cognitive functions and awareness	2.375	1.175	4.798	0.016
10-7 years before	Other symptoms and signs concerning food and fluid intake	2.284	1.417	3.684	0.001
10-7 years before	Personal history of diseases of the circulatory system	1.237	1.040	1.471	0.016
7-5 years before	Acute renal failure	0.525	0.298	0.923	0.025
7-5 years before	Bipolar affective disorder	3.869	1.555	9.631	0.004
7-5 years before	Chest pain	1.332	1.112	1.596	0.002
7-5 years before	Degenerative disease of nervous system	7.636	2.721	21.433	0.000
7-5 years before	Disorder of urinary system	1.329	1.025	1.722	0.032
7-5 years before	Dizziness and giddiness	1.669	1.010	2.758	0.046
7-5 years before	Extrapyramidal and movement disorders	3.353	1.428	7.873	0.005
7-5 years before	Nausea and vomiting	0.683	0.468	0.996	0.048
7-5 years before	Oedema	0.379	0.163	0.881	0.024
7-5 years before	Other and unspecified symptoms and signs involving cognitive functions and awareness	2.978	1.765	5.025	0.000
7-5 years before	Other symptoms and signs concerning food and fluid intake	1.915	1.284	2.856	0.001
7-5 years before	Sprain and strain of other and unspecified parts of shoulder girdle	4.178	1.143	15.280	0.031
7-5 years before	Type 2 diabetes mellitus without complications	1.295	1.048	1.600	0.017
5-2 years before	Accidental poisoning by and exposure to other and unspecified drugs, medicaments and biological substances	6.469	1.598	26.186	0.009
5-2 years before	Chest pain	1.394	1.181	1.645	0.000
5-2 years before	Degenerative disease of nervous system	17.271	8.161	36.551	0.000
5-2 years before	Delirium	3.568	1.834	6.940	0.000
5-2 years before	Depressive episode	1.387	1.045	1.840	0.024
5-2 years before	Disorder of lipoprotein metabolism	1.173	1.009	1.365	0.038
5-2 years before	Disorder of mineral metabolism	0.478	0.240	0.951	0.036
5-2 years before	Disorder of urinary system	1.344	1.063	1.700	0.013
5-2 years before	Epilepsy	1.637	1.044	2.569	0.032
5-2 years before	Extrapyramidal and movement disorders	4.257	2.001	9.055	0.000
5-2 years before	Functional intestinal disorder	1.413	1.077	1.855	0.013
5-2 years before	Medical care	0.778	0.630	0.962	0.020
5-2 years before	Nausea and vomiting	0.663	0.469	0.938	0.020
5-2 years before	Oedema	0.423	0.207	0.865	0.018
5-2 years before	Other and unspecified symptoms and signs involving cognitive functions and awareness	3.678	2.508	5.393	0.000
5-2 years before	Other symptoms and signs concerning food and fluid intake	2.458	1.767	3.420	0.000
5-2 years before	Problem related to lifestyle	1.341	1.047	1.718	0.020
5-2 years before	Procedure not carried out reason	1.220	1.017	1.462	0.032
5-2 years before	Rash and other nonspecific skin eruption	0.261	0.110	0.621	0.002
5-2 years before	Redundant prepuce, phimosis and paraphimosis	2.225	1.025	4.833	0.043
5-2 years before	Retention of urine	0.655	0.461	0.929	0.018
5-2 years before	Unknown and unspecified causes of morbidity	1.462	1.107	1.932	0.007
5-2 years before	Unspecified fall	1.692	1.099	2.606	0.017
2 years before to diagnosis	Chest pain	1.302	1.108	1.530	0.001
2 years before to diagnosis	Degenerative disease of nervous system	46.184	17.596	121.218	0.000
2 years before to diagnosis	Delirium	4.263	2.542	7.147	0.000
2 years before to diagnosis	Disorder of mineral metabolism	0.603	0.369	0.985	0.043
2 years before to diagnosis	Disorder of urinary system	1.324	1.064	1.647	0.012
2 years before to diagnosis	Dizziness and giddiness	1.477	1.013	2.154	0.043
2 years before to diagnosis	Extrapyramidal and movement disorders	4.490	2.274	8.863	0.000
2 years before to diagnosis	Functional intestinal disorder	1.355	1.031	1.781	0.030
2 years before to diagnosis	Medical care	0.733	0.603	0.892	0.002
2 years before to diagnosis	Multiple valve disease	1.638	1.001	2.680	0.049
2 years before to diagnosis	Nausea and vomiting	0.625	0.453	0.861	0.004
2 years before to diagnosis	Other and unspecified symptoms and signs involving cognitive functions and awareness	5.381	3.912	7.402	0.000
2 years before to diagnosis	Other symptoms and signs concerning food and fluid intake	2.363	1.775	3.146	0.000
2 years before to diagnosis	Personal history of allergy to unspecified drugs, medicaments and biological substances	0.833	0.728	0.953	0.008
2 years before to diagnosis	Procedure not carried out reason	1.253	1.050	1.495	0.012
2 years before to diagnosis	Syncope and collapse	1.356	1.022	1.799	0.035
2 years before to diagnosis	Unknown and unspecified causes of morbidity	1.416	1.088	1.842	0.010
2 years before to diagnosis	Unspecified diabetes mellitus without complications	1.845	1.121	3.038	0.016
2 years before to diagnosis	Unspecified fall	1.563	1.083	2.256	0.017

Table S5: Significantly associated comorbidities with Vascular Dementia cohort from logistic regression model. Significance here refers to p-value <0.05. Conditions are displayed with corresponding odds ratios and 95% confidence intervals. Conditions are displayed in alphabetical order.

Timeframe	Condition	Odds Ratio	CI Lower	CI Upper	P-Value
20-15 years before	Type 2 diabetes mellitus without complications	2.897	1.313	6.393	0.008
15-10 years before	Cerebrovascular disease	5.442	1.491	19.863	0.010
15-10 years before	Epilepsy	7.164	1.985	25.850	0.003
15-10 years before	Hypertensive diseases	1.424	1.046	1.938	0.025
15-10 years before	Stroke, not specified as haemorrhage or infarction	4.826	1.522	15.310	0.008
15-10 years before	Type 2 diabetes mellitus without complications	2.676	1.638	4.370	0.000
10-7 years before	Epilepsy	4.633	1.653	12.981	0.004
10-7 years before	Hypertensive diseases	1.405	1.080	1.830	0.011
10-7 years before	Other specified intestinal infections	4.386	1.096	17.552	0.037
10-7 years before	Personal history of diseases of the circulatory system	1.392	1.065	1.820	0.016
10-7 years before	Stroke, not specified as haemorrhage or infarction	5.847	1.787	19.139	0.004
10-7 years before	Type 2 diabetes mellitus without complications	2.809	1.934	4.079	0.000
7-5 years before	Degenerative disease of nervous system	6.411	1.698	24.213	0.006
7-5 years before	Depressive episode	1.778	1.010	3.131	0.046
7-5 years before	Epilepsy	2.496	1.077	5.786	0.033
7-5 years before	Haemorrhage from respiratory passages	2.431	1.033	5.722	0.042
7-5 years before	Hypertensive diseases	1.395	1.098	1.774	0.007
7-5 years before	Intracerebral haemorrhage	3.416	1.070	10.902	0.038
7-5 years before	Type 2 diabetes mellitus without complications	2.444	1.724	3.465	0.000
5-2 years before	Atrial fibrillation and atrial flutter	1.629	1.168	2.274	0.004
5-2 years before	Cerebral infarction	2.452	1.368	4.394	0.003
5-2 years before	Degenerative disease of nervous system	10.508	2.942	37.532	0.000
5-2 years before	Hypertensive diseases	1.396	1.092	1.784	0.008
5-2 years before	Intracerebral haemorrhage	5.173	1.994	13.421	0.001
5-2 years before	Other and unspecified symptoms and signs involving cognitive functions and awareness	4.119	1.954	8.685	0.000
5-2 years before	Personal history of allergy to unspecified drugs, medicaments and biological substances	0.667	0.506	0.879	0.004
5-2 years before	Pulmonary embolism without mention of acute cor pulmonale	0.399	0.166	0.957	0.040
5-2 years before	Recurrent depressive disorder	5.571	1.316	23.583	0.020
5-2 years before	Schizophrenia	6.034	1.559	23.353	0.009
5-2 years before	Special screening examination	1.915	1.079	3.398	0.026
5-2 years before	Transient cerebral ischaemic attack	3.403	1.310	8.840	0.012
5-2 years before	Type 2 diabetes mellitus without complications	2.138	1.547	2.954	0.000
2 years before to diagnosis	Bipolar affective disorder	5.840	1.737	19.629	0.004
2 years before to diagnosis	Cerebral infarction	2.402	1.360	4.243	0.003
2 years before to diagnosis	Cerebrovascular disease	2.244	1.151	4.375	0.018
2 years before to diagnosis	Degenerative disease of nervous system	9.720	3.524	26.809	0.000
2 years before to diagnosis	Delirium	3.804	1.394	10.376	0.009
2 years before to diagnosis	Depressive episode	2.072	1.214	3.535	0.008
2 years before to diagnosis	Mental and behavioural disorders due to use of tobacco : unspecified mental and behavioural disorder	1.811	1.018	3.223	0.043
2 years before to diagnosis	Other and unspecified agents primarily affecting the cardiovascular system	10.210	2.531	41.190	0.001
2 years before to diagnosis	Other and unspecified symptoms and signs involving cognitive functions and awareness	4.237	2.455	7.313	0.000
2 years before to diagnosis	Spondylosis	1.527	1.014	2.300	0.043
2 years before to diagnosis	Transient cerebral ischaemic attack	2.565	1.065	6.176	0.036
2 years before to diagnosis	Type 2 diabetes mellitus without complications	1.613	1.153	2.255	0.005
2 years before to diagnosis	Unknown and unspecified causes of morbidity	1.631	1.044	2.549	0.032

Table S6: Kaplan-Meier Survival analysis for Alzheimer's Disease and Vascular Dementia cohort vs. controls. Lag refers to how much earlier (in months) the dementia sub-type cohorts reached corresponding survival probabilities compared to controls. All controls were matched based on sex and age at diagnosis of dementia. All survival probabilities included. (AD: Alzheimer's Disease, VD: Vascular Dementia).

Survival Probability	AD Lag (Months)	VD Lag (Months)
0.10	20.97	28.50
0.20	14.97	23.93
0.30	15.20	24.10
0.40	14.90	22.27
0.50	15.50	21.33
0.60	13.13	16.93
0.70	7.10	9.70
0.80	5.73	6.77
0.90	3.40	3.57

Table S7: Ethnicity data for dementia and control cohorts according to their dementia diagnosis type. The control cohort's overall diagnosis here corresponds to their age- and sex-matched dementia participant.

Overall Diagnosis	Ethnicity	Dementia Cohort Count	Control Cohort Count
Alzheimer's Disease	British	3511	3562
	Irish	112	95
	Any other white background	84	91
	Caribbean	39	22
	Indian	26	27
	Other ethnic group	19	22
	Prefer not to answer	13	5
	African	12	10
	Any other Asian background	12	5
	White and Black Caribbean	3	1
	White	9	3
	Pakistani	8	6
	Bangladeshi	5	0
	Any other mixed background	4	1
	Chinese	4	11
	White and Asian	2	3
	Do not know	1	1
	White and Black African	1	5
	Black or Black British	1	0
	Mixed	1	0
	Any other black background	0	0
Vascular Dementia	British	1331	1361
	Irish	55	35
	Any other white background	29	33
	Caribbean	15	10
	Indian	16	10
	African	11	5
	Other ethnic group	9	9
	Any other mixed background	3	1
	White	3	2
	Do not know	2	0
	Any other Asian background	1	4
	Pakistani	1	2
	Prefer not to answer	1	4
	White and Asian	1	2
	White and Black African	1	2
	White and Black Caribbean	1	2
	Chinese	0	2
	Any other black background	0	0
	Bangladeshi	0	0
	Black or Black British	1	0

Supplementary Figures

International Classification of Disease (ICD-10) System Structure

- Chapter X: Diseases of the respiratory system (J00-J99)
  - Block: Other chronic obstructive pulmonary diseases (J40-J47)
    - Category: Chronic obstructive pulmonary disease (J44)
      - J44.0 – COPD with acute lower respiratory infection
      - J44.1 – COPD with acute exacerbation, unspecified
      - J44.8 – Other specified COPD
      - J44.9 – COPD, unspecified
- Other ICD-10 Chapters (I-XXII)

Figure S1: Example of ICD-10 structure for Diseases of the respiratory system (Chapter 10). Overall, ICD-10 holds a hierarchical structure beginning from Chapters, then to Blocks and to Categories. (ICD-10: International Classification of Diseases - 10th version).

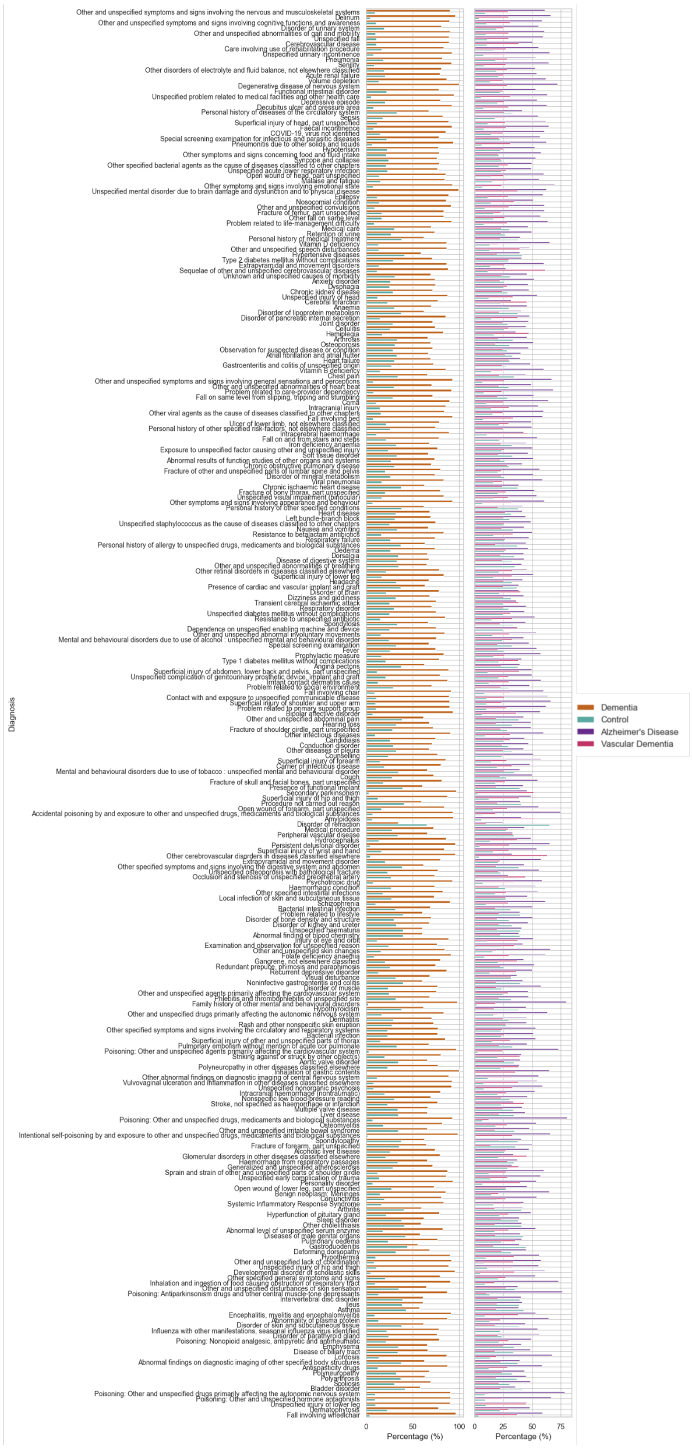

Figure S2: Prevalence of key ICD-10 conditions (individual) that were significantly different between dementia and controls. The first panel (left) is categorised by either controls or dementia cohort. The second panel (right) categorised by either controls or dementia subtypes AD and VD. Conditions are ordered from top to bottom, in order of most to least significant. Mann-Whitney U Test (all adjusted p-*value*s < 0.05, Bonferroni correction) was applied.

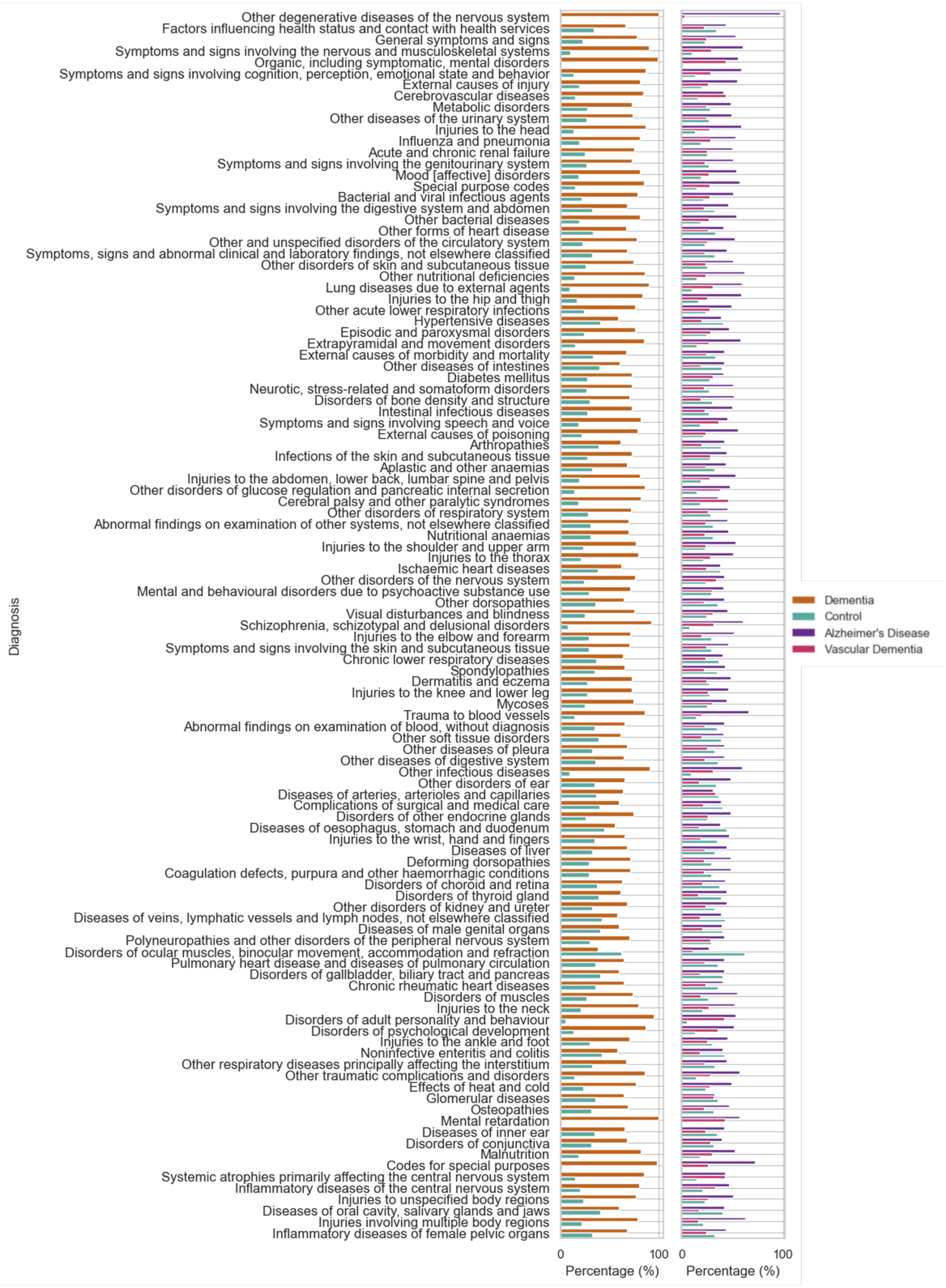

Figure S3: Prevalence of key ICD-10 conditions (blocks) that were significantly different between dementia and controls. The first panel (left) is categorised by either controls or dementia cohort. The second panel (right) categorised by either controls or dementia subtypes AD and VD. Conditions are ordered from top to bottom, in order of most to least significant. Circulatory, endocrine, bacterial/infectious, and intestinal diseases are all significantly different between the groups. Mann-Whitney U Test (all adjusted p-*value*s < 0.05, Bonferroni correction) was applied.

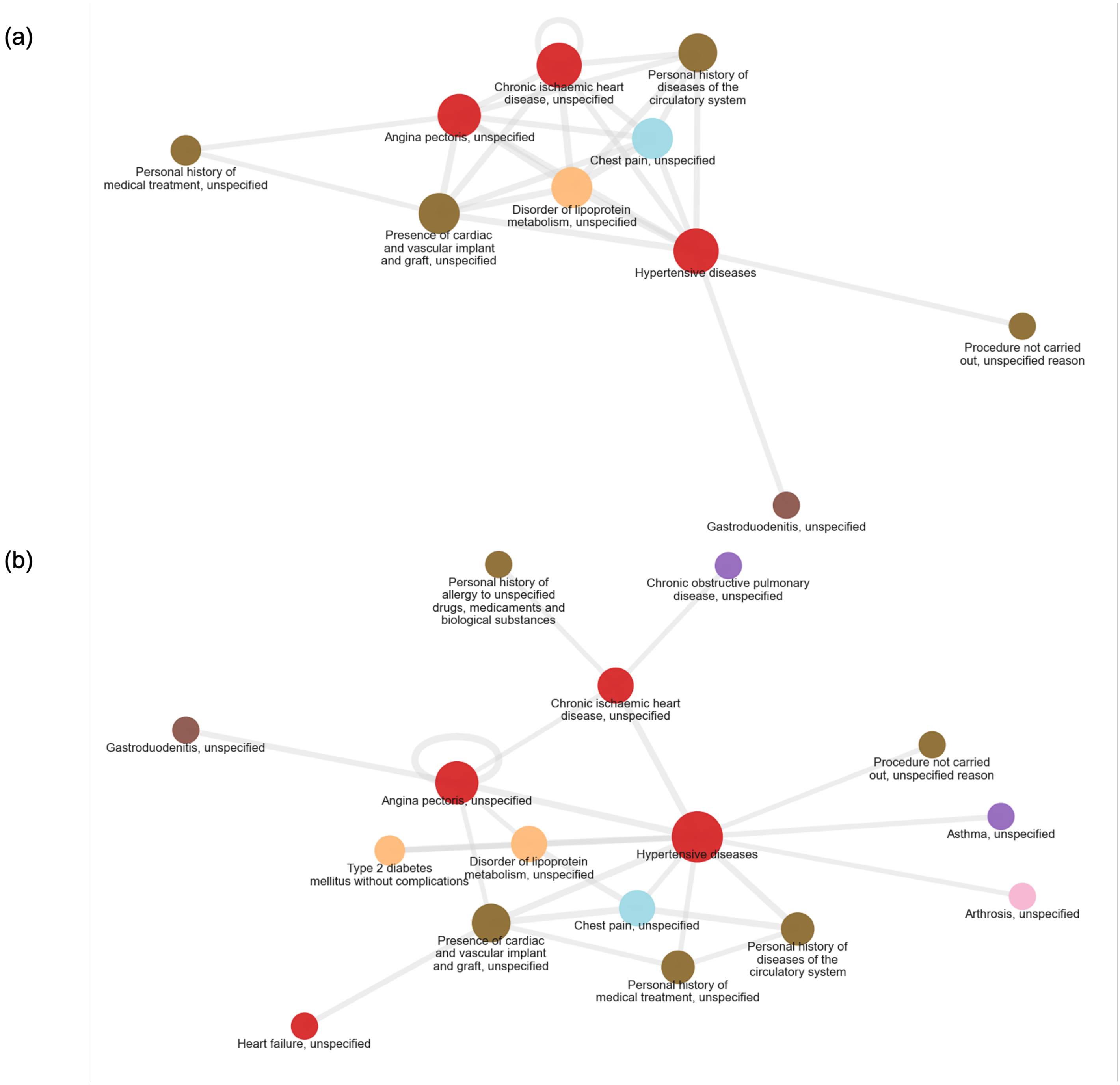

Figure S4: Network analysis showing 15-10 years before diagnosis of Alzheimer's Disease and Vascular Dementia. Panel (a) corresponds to network for AD cohort and panel (b) refers to the VD cohort. Each node (ICD-10 condition) is coloured by the corresponding ICD-10 Chapter of that condition. The size of the node corresponds to the centrality measure; the larger the node, the more centrally important and influential the condition was observed as, within each sub-type dementia group. The thickness of each edge (line between each condition) corresponds to how well-connected the condition is to other conditions in the time frame and sub-cohort. Conditions such as hypertension, circulatory disorders, diabetes and depression are common in both sub-types of dementia.

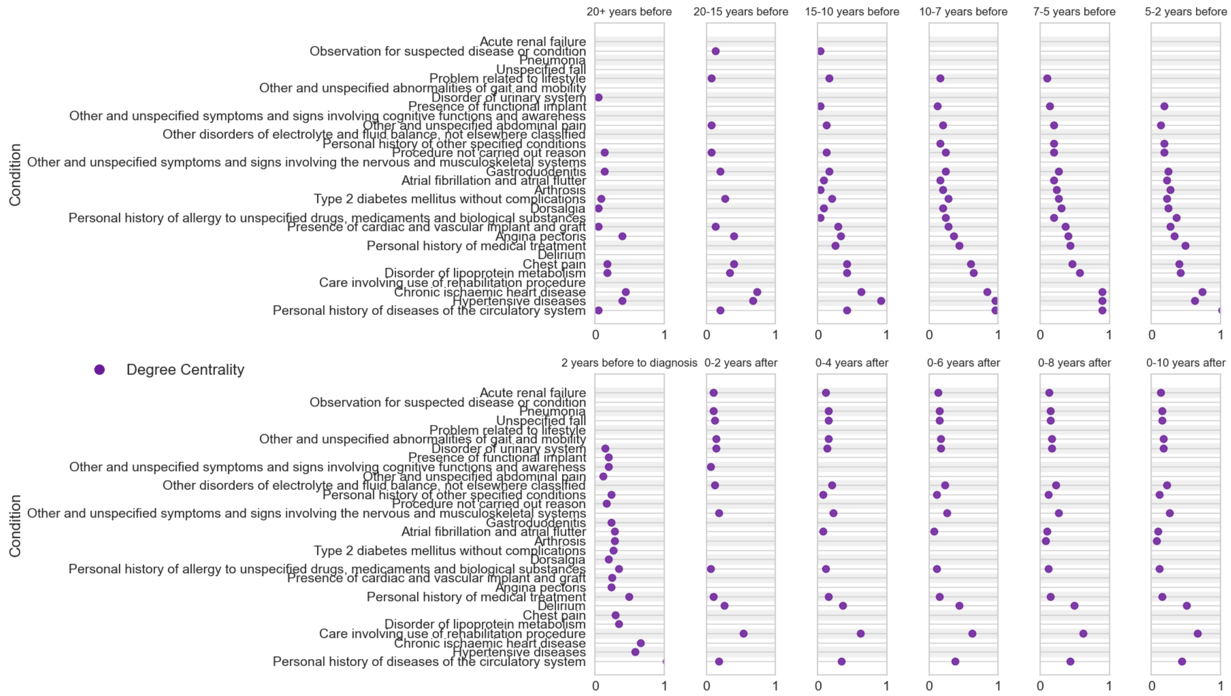

Figure S5: ICD-10 conditions with the highest centrality measures, per time frame, as measured by undirected Bayesian Network Analysis, for Alzheimer's Disease cohort. Centrality measures closer to 1.0 indicate the importance of that condition in relation to all other conditions. Hypertensive disease is seen to have high centrality at earlier time frames. Targeting conditions with higher centrality may have a more significant clinical impact.

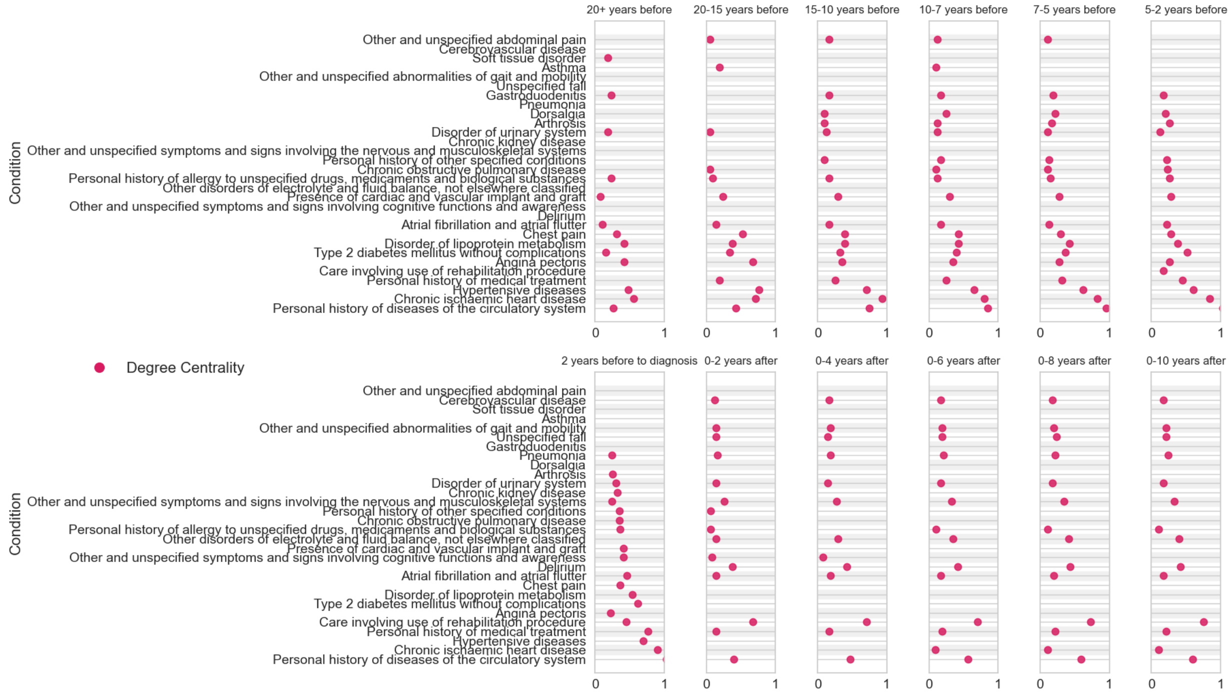

Figure S6: ICD-10 conditions with the highest centrality measures, per time frame, as measured by undirected Bayesian Network Analysis, for Vascular Dementia cohort. Centrality measures closer to 1.0 indicate the importance of that condition in relation to all other conditions. Chronic ischaemic heart disease and hypertensive disease are seen to have high centrality at earlier time frames. Targeting conditions with higher centrality may have a more significant clinical impact.

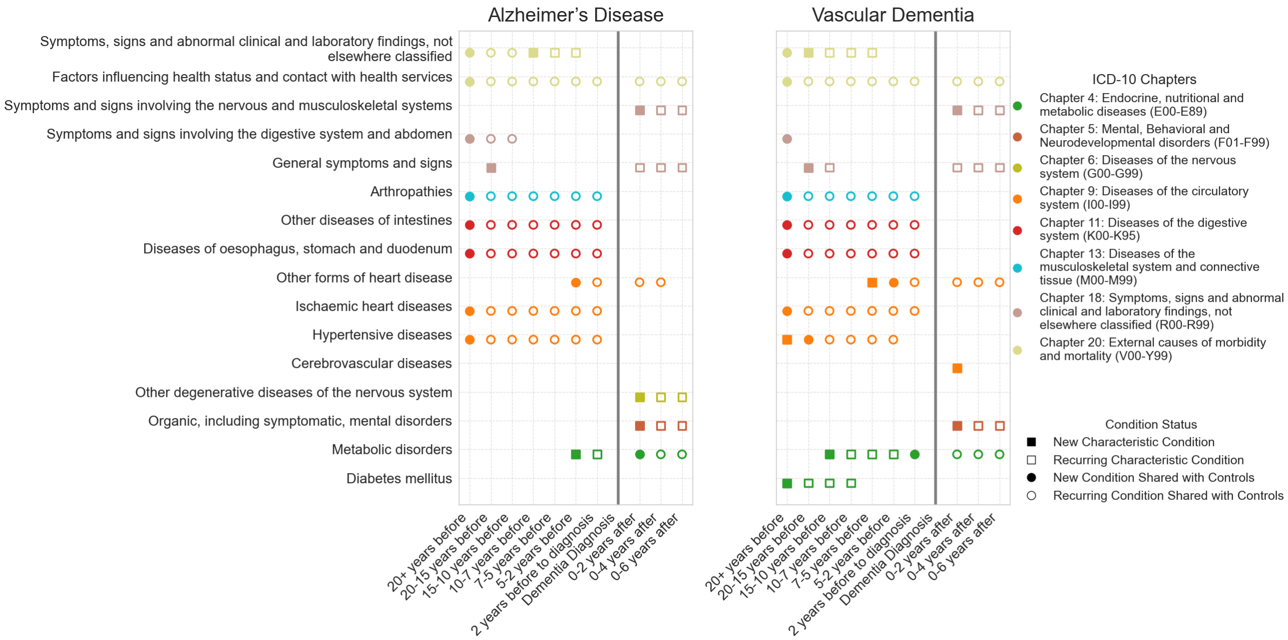

Figure S7: Chronological mapping of ICD-10 blocks for comorbidities associated with Alzheimer's Disease (left panel) and Vascular Dementia (right panel), from 20 years before diagnosis to 6 years after. Each time frame is shown, with the point of dementia diagnosis marked by a solid vertical grey line. The conditions displayed represent ICD-10 blocks or groups of comorbidities. As detailed in the first figure legend ('Condition Status'), squares represent ICD-10 blocks that were unique to the dementia sub-type cohort at the given time frame, while circles indicate blocks shared between the dementia and control cohorts. Filled shapes denote newly emerging blocks within the cohort, whereas unfilled shapes represent reoccurring blocks. Colours correspond to higher-level ICD-10 Chapters, coded in the 'ICD-10 Chapters' legend. (ICD: International Classification of Disease)

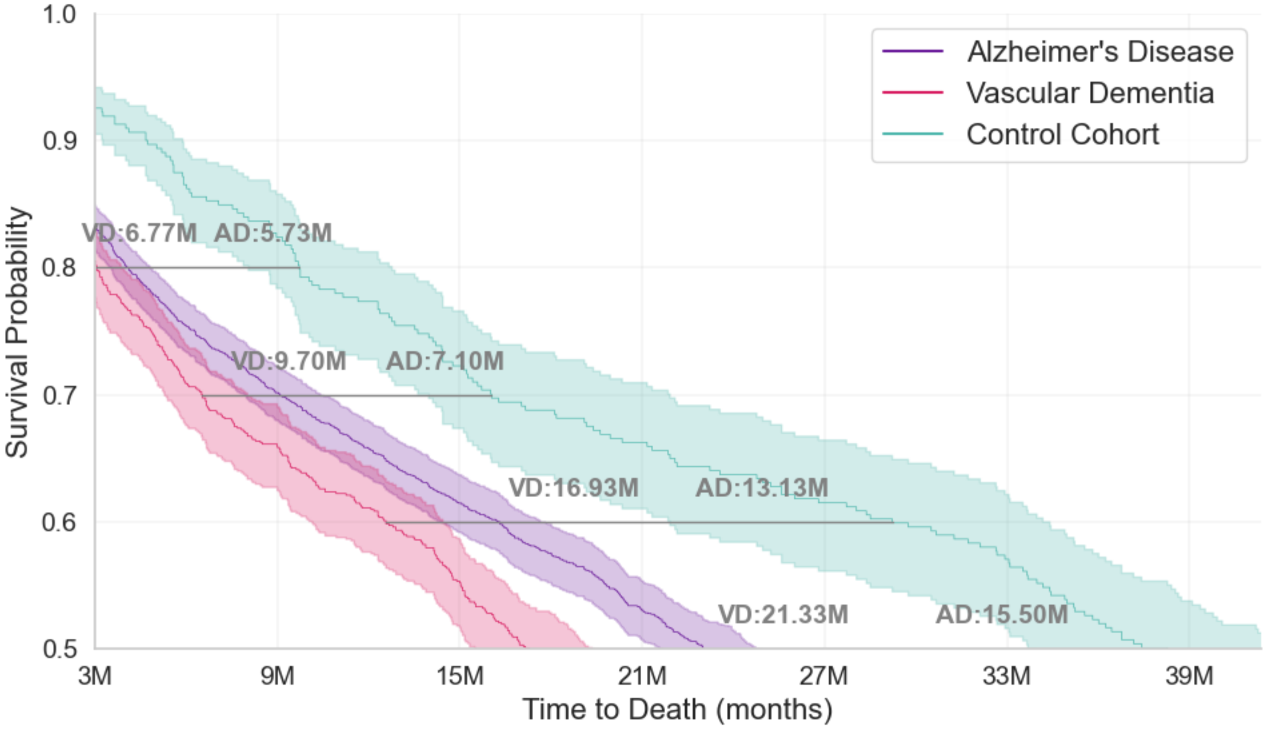

Figure S8: Kaplan-Meier Survival analysis for Alzheimer's Disease and Vascular Dementia vs. controls. Annotations show how much earlier (in months) the Alzheimer's Disease and Vascular Dementia cohorts reached corresponding survival probabilities compared to controls, up to 0.5 survival probability. All controls were matched based on sex and age at diagnosis of dementia. VD cohort showed the fastest decline overall, with more lag behind controls than the AD cohort.(AD: Alzheimer's Disease, VD: Vascular Dementia).

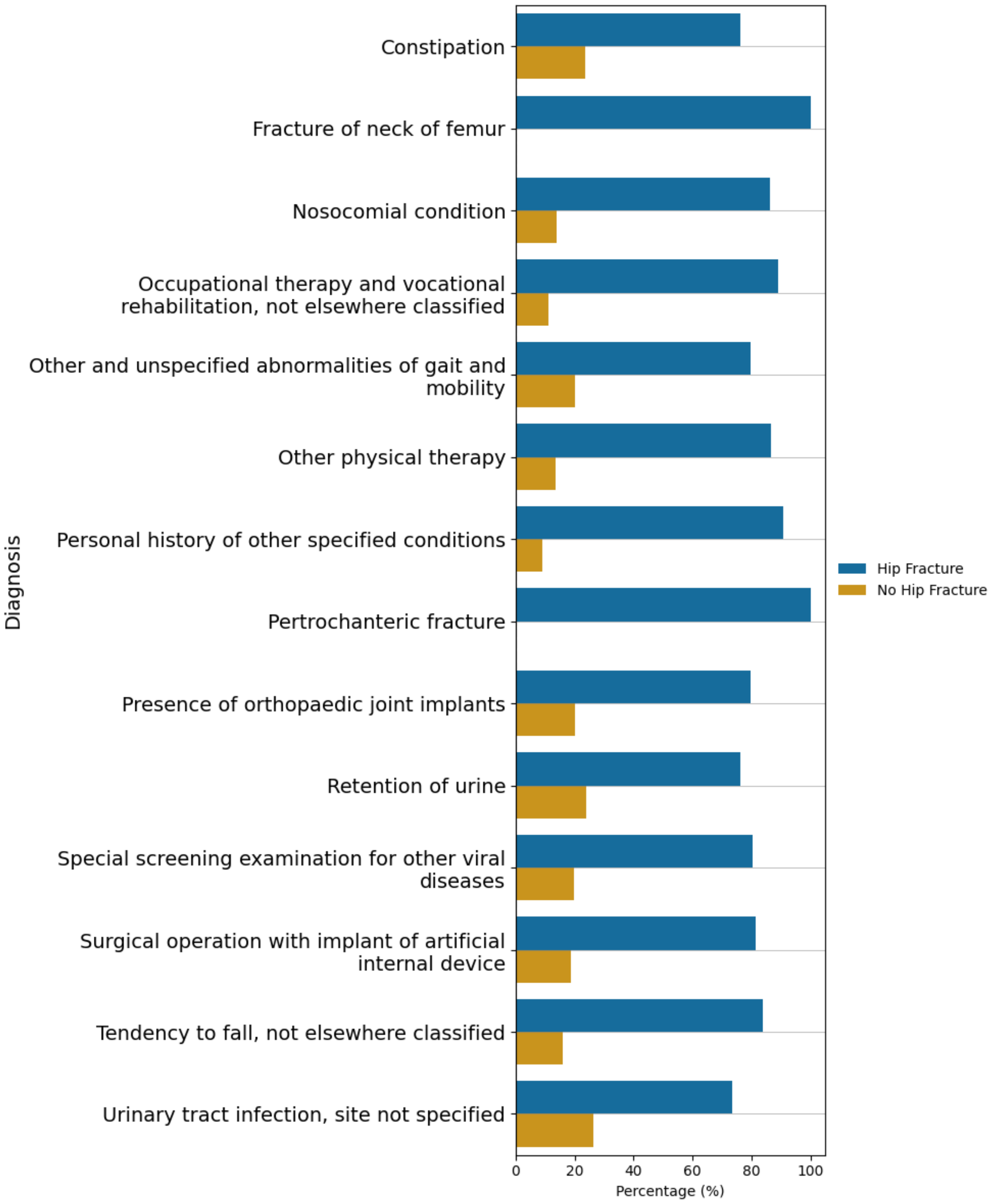

Figure S9: Prevalence of key ICD-10 conditions (individual) that were significantly different between population of people with hip fractures and controls (population without hip fractures). Conditions are ordered from top to bottom, in order of most to least significant. These conditions are not featured in the top 20 significant conditions, as with the dementia cohort and their matched controls. Mann-Whitney U Test (all adjusted p-*value*s < 0.05, Bonferroni correction) was applied.

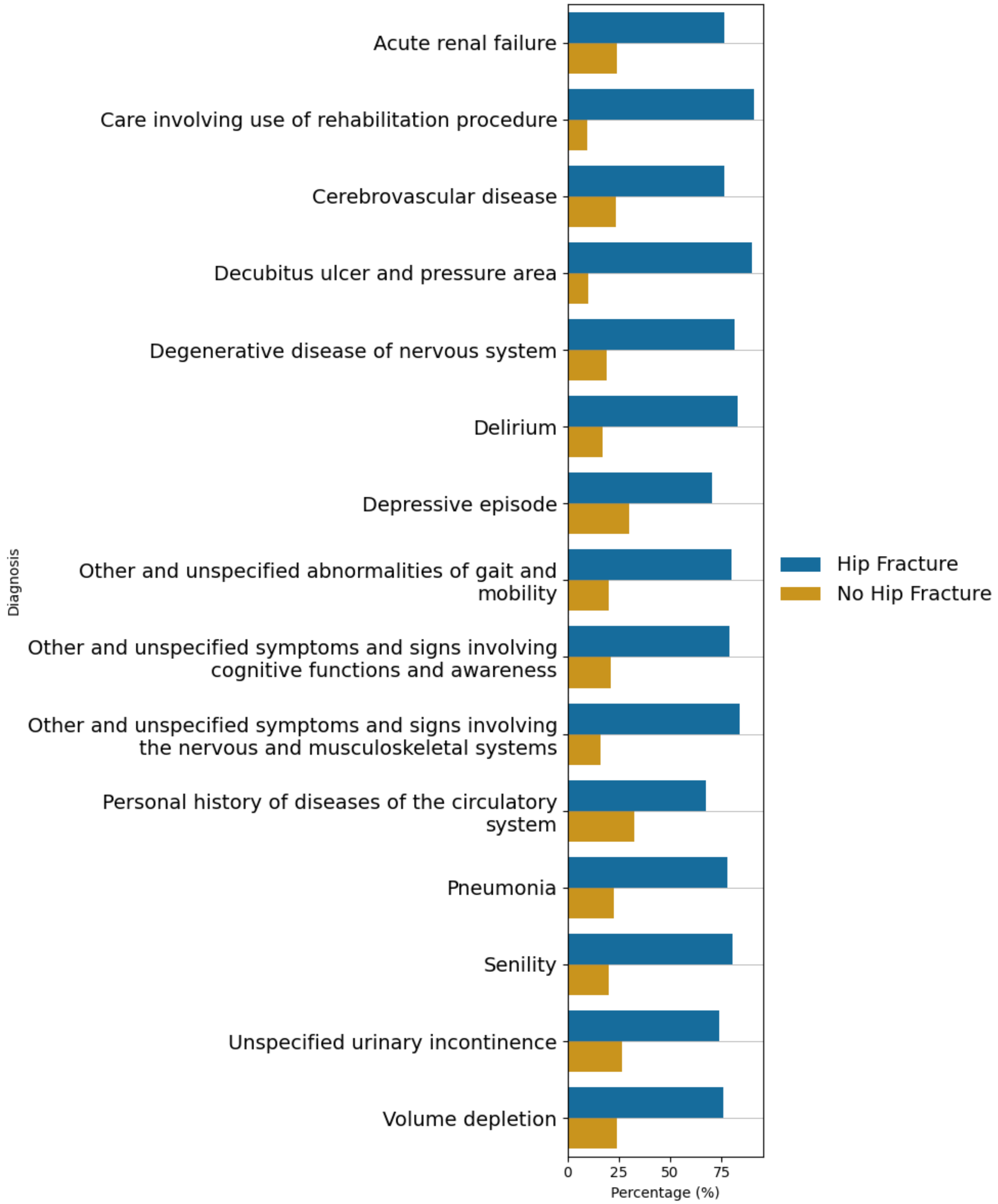

Figure S10: Prevalence of key ICD-10 conditions (individual), in the hip fracture cohort, that were identified as conditions that were significantly different between dementia and controls. Conditions are ordered from top to bottom, in order of most to least significant. The proportion of people with hip fractures are generally lower than what was seen in the dementia cohort. In addition, those without hip fractures have higher proportions than the matched controls in the dementia cohort. Mann-Whitney U Test (all adjusted p-*value*s < 0.05, Bonferroni correction) was applied.

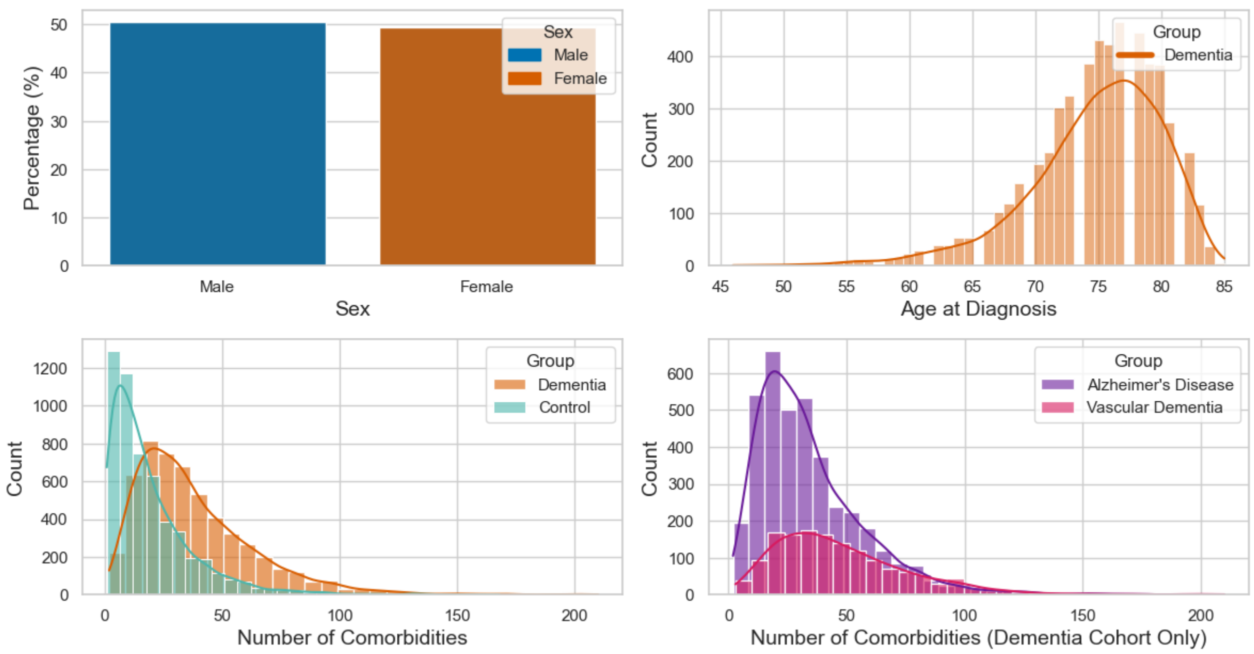

Figure S11: Baseline characteristics of dementia subtypes and control cohorts. (a) Percentage distribution of female and male participants, for dementia and control groups. (b) Distribution of age at diagnosis, for dementia cohort. (c) Polygenic risk scores for AD based on each sub-type group. The AD and VD subtype groups had significantly (adjusted p-*value* $= <$ 0.001) higher polygenic risk scores than the controls. (d) Distribution of number of diagnosed comorbidities per group for dementia and control cohorts. Dementia cohort showed on average, higher numbers of diagnosed comorbidities as compared with control cohort. (e) Distribution of number of diagnosed comorbidities per group for dementia cohort only, grouped into each sub-type, AD and VD.
